# Connecting atrial fibrillation to digestive neoplasms: exploring mediation via ischemic stroke and heart failure in Mendelian randomization studies

**DOI:** 10.3389/fonc.2024.1301327

**Published:** 2024-02-20

**Authors:** Zhijie Xu, Xuezhi Rao, Yaxuan Xing, Zhiwei Zhu, Longmei Yan, Jian Huang, Jingchun Zhang, Ruwen Zheng

**Affiliations:** ^1^ Beijing University of Chinese Medicine, Beijing, China; ^2^ Graduate School, Beijing University of Chinese Medicine, Beijing, China; ^3^ The Second School of Clinical Medicine, Beijing University of Chinese Medicine, Beijing, China; ^4^ Xiyuan Hospital, China Academy of Chinese Medical Sciences, Beijing, China; ^5^ Department of Acupuncture and Moxibustion, Dongfang Hospital, Beijing University of Chinese Medicine, Beijing, China

**Keywords:** atrial fibrillation, hypertension, heart failure, ischemic stroke, coronary artery disease, Mendelian randomization, digestive system cancers

## Abstract

**Background:**

Notwithstanding the acknowledged interplay between atrial fibrillation (AF) and the emergence of digestive system neoplasms, the intricacies of this relationship remain ambiguous. By capitalizing univariable Mendelian Randomization (MR) complemented by a mediated MR tactic, our pursuit was to elucidate the causative roles of AF in precipitating digestive system malignancies and potential intermediary pathways.

**Method:**

This research endeavor seeks to scrutinize the causal clinical implications of whether genetic predispositions to AF correlate with an increased risk of digestive system malignancies, employing MR analytical techniques. Utilizing a dataset amalgamated from six studies related to AF, encompassing over 1,000,000 subjects, we performed univariable MR assessments, employing the random-effects inverse-variance weighted (IVW) methodology as our principal analytical paradigm. Subsequently, a mediated MR framework was employed to probe the potential mediating influence of AF on the nexus between hypertension (HT), heart failure (HF), ischemic stroke (IS), coronary artery disease (CAD), and digestive system neoplasms.

**Result:**

The univariable MR evaluation unveiled a notable causal nexus between the genetic inclination toward AF and the genetic susceptibility to colon, esophageal, and small intestine malignancies. The mediated MR scrutiny ascertained that the genetic inclination for AF amplifies the risk profile for colon cancer via IS pathways and partially explains the susceptibility to esophageal and small intestine tumors through the HF pathway.

**Conclusion:**

Our investigative endeavor has highlighted a definitive causative association between genetic inclination to AF and specific digestive system neoplasms, spotlighting IS and HF as instrumental mediators. Such revelations furnish pivotal perspectives on the complex genetic interconnections between cardiovascular anomalies and certain digestive tract tumors, emphasizing prospective therapeutic and diagnostic worthy of pursuit.

## Introduction

Atrial fibrillation (AF), the predominant cardiac dysrhythmia, fluctuates between symptomatic and asymptomatic manifestations. Given its escalating prevalence, there is a critical need for efficacious prophylactic interventions (1, 2). The sheer breadth of the afflicted demographic poses a substantial challenge to public health, underscored by the multifaceted sequela of AF—including ischemic stroke (IS), heart failure (HF), coronary artery disease (CAD), cognitive declination, and heightened mortality (3, 4). Intriguingly, an AF diagnosis heralds an augmented risk of malignancy (5). Cancer incidence in AF patients outstrips that of the broader populace by a staggering 30–40% (6). Numerous investigations posit that AF’s onset may intimate concealed neoplasms, especially pronounced within the initial trimester post-diagnosis. A Danish longitudinal study intimated that nascent AF diagnoses correlate with a magnified cancer proclivity within the inaugural three months (7). Scrutiny of the Women’s Health Study echoed this sentiment, identifying a surge in oncological susceptibility post-AF diagnosis, notably acute within its early stages. In alignment with these observations, an investigation based on the Danish Diet, Cancer, and Health study identified an elevated cancer risk—across genders—within the initial 90 days following AF diagnosis, notably with a prominence in colorectal malignancies that persists throughout prolonged observational periods (8, 9). While prior observational analyses corroborate AF’s association with heightened digestive system cancer risks, the causal nexus remains enigmatic. Concomitantly, AF frequently coexists with other cardiovascular pathologies, including HT, HF, IS, and CAD (10, 11). A retrospective analysis involving 332,555 AF patients without prior cancer histories unveiled notable rates of comorbidities: 68.5% with HT, 41.1% with HF, and 34.9% with a history of IS (6). These coexisting cardiovascular maladies are not mere bystanders; they are implicated as risk catalysts for diverse cancers. Consequently, we hypothesize that cardiovascular afflictions, spanning HT, HF, IS, and CAD, could potentially bridge the nexus between atrial fibrillation and digestive system malignancies.

Within the complex framework of genetic analyses, MR arises as a sophisticated methodology, grounded in genetic variants, providing a means to elucidate causal relationships under specified principles (12, 13). Traditionally, MR investigates the relationships between distinct exposures and their resulting outcomes in depth (14–16). The two-step MR employs an innovative strategy, grounded in the established MR framework, augmenting the accuracy of causal mediation understanding. Through this approach, one can delineate the causal impact of an exposure on an outcome, either in isolation (direct effect) or mediated via an intermediary (indirect effect) (17, 18). Notably, the two-step Mendelian randomization (MR) approach eliminates the necessity for detailed individual-level data, opting instead for the utilization of genome-wide association studies (GWAS) summary statistics. These statistics are frequently derived from extensive population samples, encompassing a diverse range of traits and phenotypes (19).

In our scholarly pursuit, human genetics were leveraged to hypothesize a causal association between AF and the susceptibility to a septet of digestive system malignancies. This investigation initiated an exhaustive exploration of traits associated with AF. Subsequently, causal mediation analyses, supported by the two-step MR approach, were rigorously employed to elucidate the potential intermediary roles of HT, HF, IS, and CAD in the causative linkage with these digestive system malignancies.

## Materials and methods

### Study design

**Figure 1:**
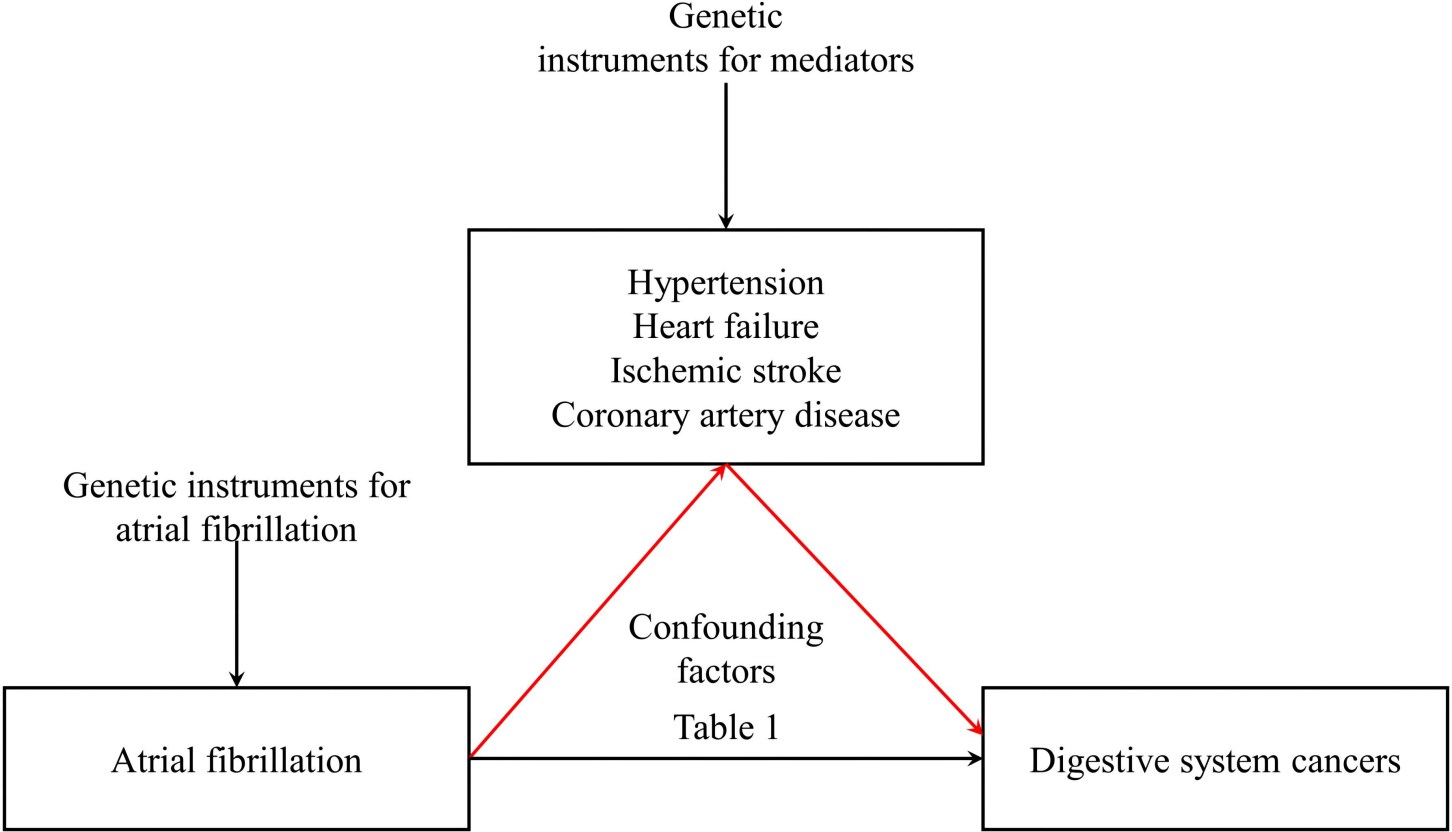
Observational and genetic associations/mediations investigated in the present study.

This investigation commenced with summary-level data obtained from GWAS focusing on traits associated with AF (20). Extending previous epidemiological inquiries, we performed a two-sample MR analysis concerning atrial fibrillation and malignancies of the digestive system. Subsequently, through the robust framework of two-step MR, causal mediation analyses were utilized to shed light on the potential intermediary roles of HT, HF, IS, and CAD in establishing a causative connection with these gastrointestinal neoplasms. (refer to **Figure 1**) This MR investigation adheres to the subsequent three foundational assumptions: 1. Pertinence: Demonstrating a noteworthy association with the exposure in question at a significance level of p < 5 × 10^−8^, the genetic variant maintains pertinence. 2. Independence: The genetic variant persists independently, showing no association with any confounding factors that could potentially distort the exposure-outcome relationship, as evidenced by the horizontal pleiotropy test. 3. Restriction of Exclusion: Exerting influence on the outcome exclusively through its potential ramifications on the exposure under examination, the genetic variant has been verified by consulting PhenoScanner V2.

### Data sources

#### (1) Exposure GWAS

We utilized genome-wide summary statistics from a published meta-analysis on AF, which included six study cohorts (20). The Nord-Trøndelag Health Study (HUNT) is an enduring, population-based health survey, steadfastly conducted in Norway since 1984 (21). The deCODE study encompasses all patients diagnosed with atrial fibrillation at Landspitali from 1987 to 2015, encapsulating the entirety of the Icelandic atrial fibrillation populace. The Michigan Genomics Initiative represents a hospital-based cohort accrued in the United States. The DiscovEHR incorporates a hospital-based assemblage of European lineage in the United States (22). The UKBB is a population-based cohort in the United Kingdom (23). The AFGen Consortium proffers atrial fibrillation association summary statistics derived from 31 cohorts. In total, these studies represent a significant cohort of 60,620 cases and 970,216 controls (24).

#### (2) Mediator GWAS

The CAD and HF data were from the archives of the UK Biobank (UKBB), including 34,541 cases and 261,984 controls for CAD (25), 1,405 cases, and 359,789 controls for HF. The IS data was from a genome-wide association study comprising 7,193 cases and 406,111 controls (26) and HT data was from the International Consortium of Blood Pressure comprising 757,601 individuals (27).

#### (3) Outcome GWAS

The FinnGen consortium constitutes a research initiative that aggregates health and genetic data derived from Finnish health registries (28). It operates as a public–private partnership research project, amalgamating imputed genotype data obtained from both newly collected and legacy samples sourced from Finnish biobanks, alongside digital health record information sourced from Finnish health registries (https://www.finngen.fi/en). The project harnesses data from the nationwide longitudinal health register, spanning data collection since 1969 and encompassing every resident in Finland (29). These 7 digestive system cancers were from cancer register (ICD-O-3) of FinnGen studies (29): the case group comprised 126 cases of cancers in the lip, oral cavity, and pharynx, 232 cases of esophageal cancers, 633 cases of stomach cancers, 605 cases of pancreas cancers, 252 cases of small intestinal cancers, 1803 cases of colorectal cancers, and 1078 cases of rectal cancers. The control group for all the aforementioned cancers consisted of 174,006 individuals. It is imperative to note that the current analysis harnesses exclusively publicly accessible summary statistics, thus circumventing the necessity for additional ethical consent.

### Selection of IVs for MR analyses

**Figure 2:**
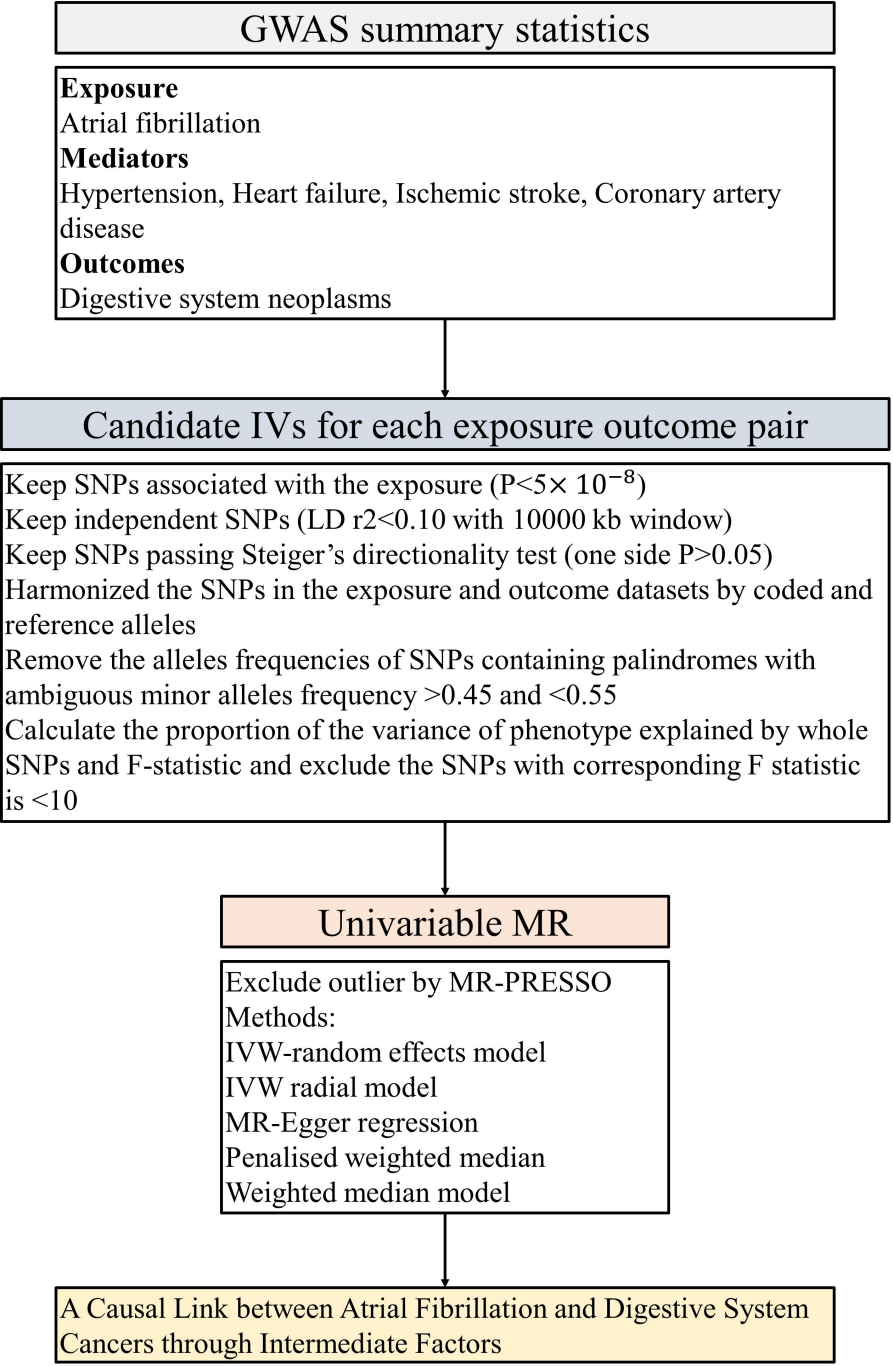
The flow chart of the inclusion and exclusion criterion of candidate SNPs for each exposure-outcome pair. GWAS, genome-wide association studies; LID, Linkage disequilibrium; IVW, inverse-variance weighted; PRESSO, Pleiotropy Residual Sum and Outlier; MR, Mendelian randomization.

In this investigation, we scrutinized the following traits: AF, CAD, HF, IS, and HT. MR analyses were conducted among individuals of European descent, employing genome-wide significance (p < 5 × 10^−8^) and linkage disequilibrium (R^2^ <0.1) subsequent to standard exclusions (20). These exclusions encompass instances of withdrawn consent, suspected sex chromosome aneuploidy, and discrepancies between genetically inferred and self-reported gender (30). Appropriate instrumental variables (IVs) for the MR evaluations were culled from disparate GWAS summary findings. Single-nucleotide polymorphisms (SNPs) that met the rigorous criterion of genome-wide significance (p < 5 × 10^−8^) were selected during the initial phase. Subsequently, relevant SNPs were retained based on the linkage disequilibrium criterion, stipulated by an R² < 0.1 according to the Genome reference panel (31). SNPs exhibiting an association with the outcome variables at a significance level of p < 5 × 10^−8^ were methodically excluded from consideration. IVs signifying correlations with AF, CAD, HF, IS, and HT-associated characteristics, meeting conventional GWAS thresholds (P < 5 × 10^−8^), were selected for each respective phenotype, refer to **Figure 2**. A genetic instrument embodies one or numerous genetic variances imbued with attributes conducive to their utilization as an IV within the purview of MR (12). Throughout the harmonization process encompassing both exposure and outcome data sets, palindromic SNPs and those devoid of requisite information were removed. The robustness of the IVs was evaluated via the computation of F-statistics, with values beneath the threshold of 10 indicating an inherently weak instrument strength, thereby necessitating their removal from the analysis (32, 33).

### MR analysis

Initially, we executed univariable MR analyses for AF on 7 digestive system cancers. To amalgamate an aggregate effect estimate across a multitude of genetic instruments, we employed an array of four distinct methodologies: inverse-variance weighted (IVW), the weighted median method, MR-Egger, and weighted mode (34). After this, we sought to tackle the exclusion restriction assumption by employing the MR-Egger regression intercept technique. This method assessed potential biases in causal estimations arising from the complex issue of horizontal pleiotropy, considering its associated 95% confidence interval (CI) (35, 36). Furthermore, we assessed the presence of horizontal pleiotropy using the MR pleiotropy residual sum. Simultaneously, outlier SNPs were identified and removed using the MR-PRESSO outlier test (p < 0.05), thereby ensuring the robustness and integrity of the analysis (37). We conducted an investigation of the pleiotropic implications of each genetic instrument by consulting PhenoScanner V2 to identify secondary phenotypes associated (p < 5 × 10^−8^) with the IVs or their proxies (r² > 0.8) (38). It is crucial to explicate the importance of incorporating multiple testing as an essential statistical methodology. However, in the context of this article, structured as an exploratory study with the objective of unveiling a diverse range of potential positive associations, and considering the utilization of non-identical SNPs in each two-sample MR study, we have temporarily abstained from implementing multiple testing (39). Acknowledging the significant roles of HT, HF, IS, and CAD as consequential outcomes of AF, we aimed to determine whether the causative impacts of AF on these malignancies pass through these mediators (40). The proportionality of this mediated effect was ascertained by comparing the indirect effect to the overall effect. The mediating proportion was epitomized as the quotient of the indirect to the comprehensive effect, and was deemed “non-causal” should the total and indirect effects diametrically oppose.

We incorporated all GWAS-associated SNPs for each trait into the model and calculated them in four ways. We demarcated causal estimates for binary outcomes, complemented by p-values for each distinct methodology, β coefficients, and their corresponding standard errors. It is quintessential to acknowledge that all p-values were ascertained by employing a two-tailed paradigm.

### Sensitivity analyses

In the field of sensitivity analyses, we performed univariable MR assessments that were robust to specific manifestations of potential unbalanced horizontal pleiotropy. This involved the utilization of MR-Egger methodologies. In the realm of sensitivity analyses, we engaged in univariable MR evaluations resilient to certain manifestations of potential unbalanced horizontal pleiotropy (41), utilizing weighted median (42), weighted mode (43), and MR-Egger methodologies (35). We conducted a variety of sensitivity analyses, encompassing leave-one-out assessment and single SNP analysis, aimed at determining if an individual SNP was exerting a disproportionate sway on the primary causal relationship under scrutiny (36).

To conduct these analyses, we harnessed the MendelianRandomization and TwoSampleMR packages within the R programming milieu (version 4.2.0, available at www.r-project.org/).

## Results

### Univariable MR analysis of AF on seven digestive system cancers

**Figure 3:**
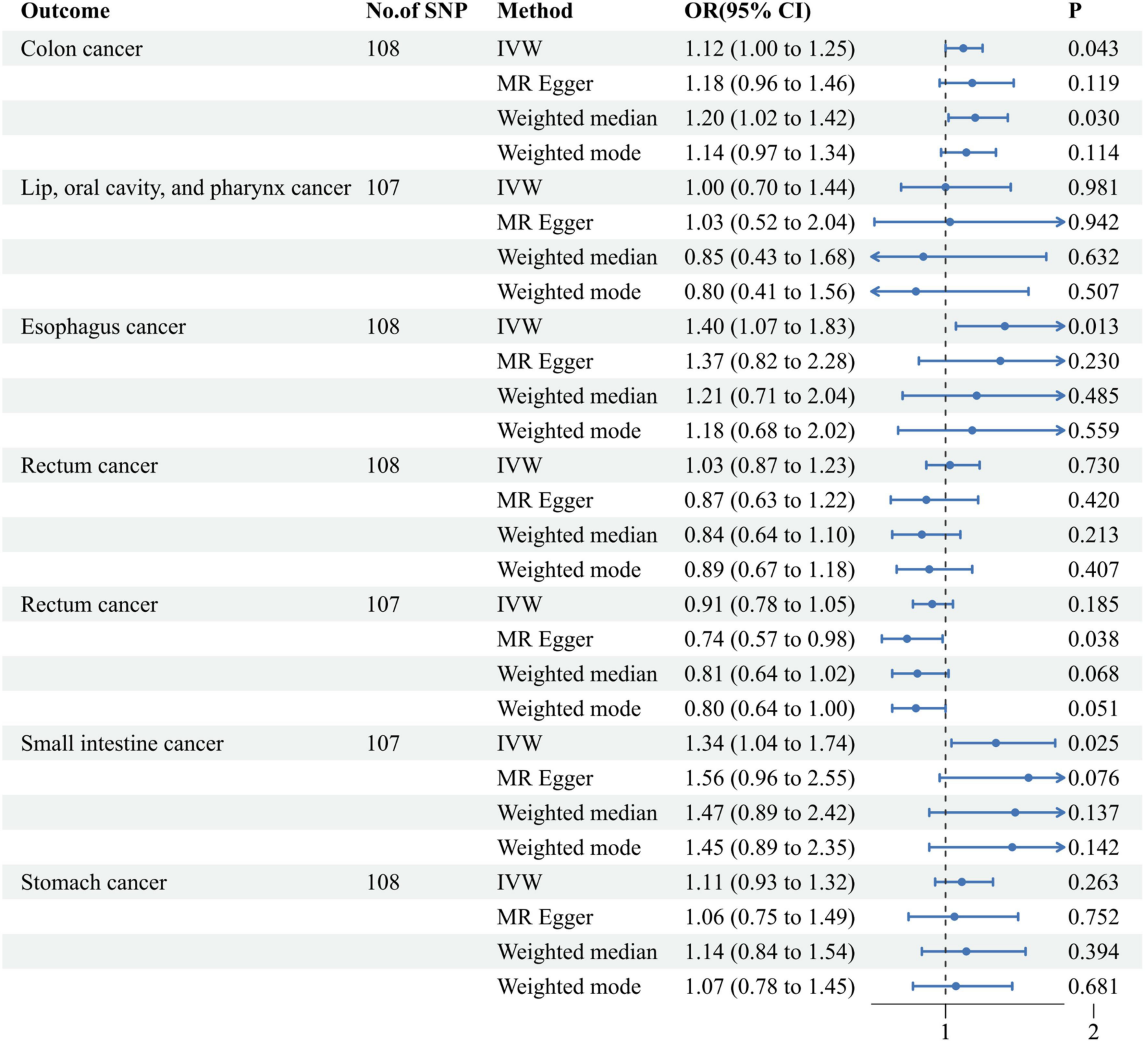
Associations of genetically predicted atrial fibrillation with seven digestive system cancers. IVW, inverse-variance weighted; MR, Mendelian Randomization; Cl, confidence interval; OR, Odds ratio; SNP, single nucleotide polymorphism.

The univariable MR analysis elucidated that a genetically deduced susceptibility to AF bears a robust causal correlation with the genetic inclinations for colon cancer (IVW approach: Odds Ratio (OR) [95% CI] = 1.12 [1.00, 1.25], p = 0.043), esophageal cancer (IVW approach: OR [95% CI] = 1.40 [1.07, 1.83], p = 0.013), and small intestine cancer (IVW approach: OR [95% CI] = 1.34 [1.04, 1.74], p = 0.025). However, MR evaluations of the ensuing tetrad of malignancies revealed no pronounced causal ties: neoplasms of the lip, oral cavity, and pharynx (IVW approach: OR [95% CI] = 1.00 [0.70, 1.44], p = 0.981), pancreatic cancer (IVW approach: OR [95% CI] = 1.03 [10.87, 1.23], p = 0.730), rectal cancer (IVW approach: OR [95% CI] = 0.91 [0.78, 1.05], p = 0.185), and gastric cancer (IVW approach: OR [95% CI] = 1.11 [0.93, 1.32], p = 0.263). (refer to **Figure 3**) Complementarily, insights derived from the weighted median technique, MR-Egger, and weighted mode resonated harmoniously with the IVW determinations, accentuating the rigor of our analytical paradigm (refer to **Supplementary Table 2**). A comprehensive delineation of the SNPs associated with AF traits is furnished in **Supplementary Table 1**.

### Assessing the potential mediation of digestive system cancers by AF via HT, HF, IS, or CAD

In light of the substantiated nexus between AF and ailments such as HT, HF, IS, and CAD, we endeavored to ascertain whether the interrelation of digestive system neoplasms with AF is orchestrated via these cardiovascular afflictions (40). Firstly, we validated the relationship between atrial fibrillation and these mediating factors, establishing the existence of varying degrees of causal linkage among them (refer to **Supplementary Figure 1**). Through MR mediation analyses, we inferred that the genetic predilection toward AF amplifies the peril of colon cancer through ischemic stroke mechanisms, manifesting an effect magnitude of 1.12 (95% CI: 1.00-1.25, p = 0.039). Furthermore, MR mediation analyses revealed that genetic predispositions to AF partially contribute to the development of esophageal and small intestine neoplasms through HF pathways. The observed effect sizes were 1.36 (95% CI: 1.11-1.70, p = 0.006) with 92% of the attributable risk of genetically predicted AF for esophageal cancer and 1.28 (95% CI: 1.04-1.56, p = 0.017) with 83% of the attributable risk for small intestine cancer. In contrast, other examined outcomes exhibited no discernible divergences (refer to **Table 1**). Given prior evidence demonstrating bidirectional causation between AF and HF (44), we supplemented our analysis with a two-sample MR study investigating the causal relationship between HF and digestive system cancers. The results indicate a lack of significant causal association (refer to **Supplementary Figure 1**).

**Table 1:** Mediation analysis of AF on digestive system cancers through HT, HF, IS, or CAD.

Cancer	Mediator	Total Effect(OR)	Direct Effect(OR)	Mediation Effect(OR)	
		Effect size (95% CI)	Effect size (95% CI)	Effect size (95% CI)	P
Colon cancer	SBP	1.12(1.00,1.25)	1.12(1.00, 1.25)	1.00(0.99, 1.00)	0.519
	DBP	1.12(1.00,1.25)	1.12(1.00, 1.25)	1.00(0.99, 1.00)	0.711
	HF	1.12(1.00,1.25)	1.12(0.98, 1.28)	1.00(0.93, 1.07)	0.98
	Ischemic Stroke	1.12(1.00,1.25)	0.98(0.82, 1.16)	1.12(1.00, 1.25)	0.039
	CAD	1.12(1.00,1.25)	1.14(1.02, 1.28)	0.98(0.97, 1.00)	0.029
Lip, oral cavity, and pharynx cancer	SBP	1.00 (0.70,1.44)	1.00(0.70, 1.44)	1.00(0.98, 1.02)	0.76
	DBP	1.00 (0.70,1.44)	1.00(0.70, 1.44)	1.00(0.99, 1.01)	0.964
	HF	1.00 (0.70,1.44)	0.95(0.61, 1.48)	1.06(0.82, 1.37)	0.663
	Ischemic Stroke	1.00 (0.70,1.44)	1.01(0.55, 1.85)	0.99(0.61, 1.61)	0.977
	CAD	1.00 (0.70,1.44)	1.01(0.70, 1.45)	1.00(0.94, 1.05)	0.917
Esophagus cancer	SBP	1.40(1.07,1.83)	1.40(1.07, 1.83)	1.00(0.99, 1.01)	0.555
	DBP	1.40(1.07,1.83)	1.40(1.07, 1.83)	1.00(0.99, 1.01)	0.674
	HF	1.40(1.07,1.83)	1.03(073, 1.46)	1.36(1.11, 1.70)	0.006
	Ischemic Stroke	1.40(1.07,1.83)	1.05(0.63, 1.75)	1.33(0.86, 2.06)	0.194
	CAD	1.40(1.07,1.83)	1.46(1.12, 1.92)	0.96(0.91, 1.00)	0.089
Pancreas cancer	SBP	1.03(0.87,1.23)	1.03(0.87, 1.23)	1.00(0.99, 1.01)	0.659
	DBP	1.03(0.87,1.23)	1.03(0.87, 1.23)	1.00(0.99, 1.01)	0.755
	HF	1.03(0.87,1.23)	1.02(0.83, 1.26)	1.01(0.89, 1.13)	0.905
	Ischemic Stroke	1.03(0.87,1.23)	1.14(0.87, 1.53)	0.90(0.72, 1.12)	0.333
	CAD	1.03(0.87,1.23)	1.04(0.87, 1.24)	0.99(0.96, 1.02)	0.418
Rectum cancer	SBP	0.91(0.78,1.05)	0.91(0.78, 1.05)	1.00(0.99, 1.01)	0.943
	DBP	0.91(0.78,1.05)	0.91(0.78, 1.05)	1.00(0.99, 1.00)	0.843
	HF	0.91(0.78,1.05)	0.92(0.77, 1.09)	0.99(0.91, 1.08)	0.818
	Ischemic Stroke	0.91(0.78,1.05)	1.09(0.87, 1.37)	0.83(0.70, 0.98)	0.03
	CAD	0.91(0.78,1.05)	0.92(0.97, 1.06)	0.99(0.97, 1.01)	0.344
Small intestine cancer	SBP	1.34(1.04,1.74)	1.34(1.04, 1.73)	1.00(0.99, 1.01)	0.487
	DBP	1.34(1.04,1.74)	1.35(1.04, 1.74)	1.00(0.99, 1.01)	0.657
	HF	1.34(1.04,1.74)	1.05(0.76, 1.46)	1.28(1.04, 1.56)	0.017
	Ischemic Stroke	1.34(1.04,1.74)	0.95(0.62, 1.46)	1.41(1.00, 2.00)	0.05
	CAD	1.34(1.04,1.74)	1.34(1.03, 1.73)	1.01(0.96, 1.05)	0.802
Stomach cancer	SBP	1.11(0.93,1.32)	1.11(0.93, 1.32)	1.00(0.99, 1.01)	0.932
	DBP	1.11(0.93,1.32)	1.11(0.93, 1.32)	1.00(0.99, 1.01)	0.76
	HF	1.11(0.93,1.32)	1.11(0.84, 1.48)	1.00(0.80, 1.24)	0.967
	Ischemic Stroke	1.11(0.93,1.32)	1.13(0.82, 1.57)	0.98(0.74, 1.28)	0.86
	CAD	1.11(0.93,1.32)	1.12(0.93, 1.34)	0.99(0.96, 1.02)	0.609

AF, atrial fibrillation; HT, hypertension; SBP, systolic blood pressure; DBP, Diastolic blood pressure; HF, heart failure; IS, ischemic stroke; CAD, coronary artery disease; OR, odds ratio; CI, confidence interval.

### Sensitivity analyses

In the context of the univariable MR evaluation, the outcomes gleaned from sensitivity analyses proffered no tangible evidence suggestive of latent horizontal pleiotropy as discerned through the MR-Egger intercept regression evaluations (Intercept < 0.01, p > 0.05, **Supplementary Table 2**). However, the p-value resulting from Cochran’s Q test, which integrates information from both the IVW and MR-Egger methodologies (p < 0.05, **Supplementary Table 2**), revealed significant heterogeneity in certain univariable MR analyses. Scatter diagrams vividly illustrated the potential causal relationships connecting AF and these digestive malignancies, as interpreted through the four MR methodologies employed (refer to **Supplementary File 1**). The steadfastness of our discoveries was further buttressed by the unwavering nature of the delineated associations, even upon the excision of any singular SNP in ‘leave-one-out’ explorations (refer to **Supplementary File 2**) or in isolated SNP assessments (refer to **Supplementary File S3**).

## Discussion

In this study, we performed an MR investigation to clarify the potential causal nexus between AF and the susceptibilities of seven digestive malignancies. Simultaneously, we explored the potential mediating roles of HT, HF, IS, and CAD within a large AF cohort comprising over a million individuals from six distinct studies. The evidence suggests that AF is positively correlated with the likelihood of esophageal, colon, and small intestine malignancies. Importantly, ischemic stroke acts as a mediator for colon cancer, while heart failure plays a similar role for both esophageal and small intestine cancers (see **Figure 3**; **Supplementary Table 2**). These findings confirm a tangible causal link between a genetic predisposition for AF and specific digestive neoplasms, emphasizing the importance of preventing ischemic strokes and heart failures in individuals with AF. Meanwhile, it is well-established that specific digestive malignancies heighten the risk of AF (45). Moreover, AF is unequivocally identified as a pronounced risk factor for certain digestive cancers. Previous research reveals that one in four individuals diagnosed with atrial fibrillation has a preceding cancer diagnosis (46), and extensive cohort studies demonstrate an increased likelihood of cancer detection within the initial three months following the onset of the inaugural atrial fibrillation episode (5, 7).

To our current comprehension, extant MR investigations have not scrupulously delineated the interplay between AF and digestive malignancies, including the potential intermediary pathways. Nevertheless, numerous intricate biological processes are hypothesized to explain the association, highlighting the significance of AF in the onset of susceptibility to digestive cancer. Research indicates a notable prevalence of digestive malignancies in individuals with AF (47–49). One plausible conjecture suggests that anticoagulant administration in AF therapy may reveal latent malignancies by provoking hemorrhagic events within the neoplasm (50). Furthermore, the interconnection between inflammation in the genesis of cardiovascular ailments and oncogenesis is a dynamic area of investigation, recurring across these maladies. Increased synthesis of chemotactic agents and cytokines, specifically interleukins 1 and 6, along with systemic acute-phase reactants like C-reactive protein (CRP), has been delineated in individuals afflicted with digestive malignancies (51). Concurrently, inflammatory indices, including elevated leukocyte counts, and CRP, correlate with an amplified propensity to manifest incipient (52) and pronounced AF, potentially via instigating structural and electrical atrial alterations (53). In an encompassing survey involving 5,806 participants, an ascended CRP index correlated with prevalent AF and prognosticated forthcoming episodes of this dysrhythmia (54). Intriguingly, inflammation may potentiate AF and oncogenesis via reactive oxygen species (ROS) — by-products of metabolic processes and oxygen utilization. ROS are intrinsically linked to elevated oncogenic risk due to DNA impairment and genetic flux (55, 56). In AF, ROS genesis, mediated by leukocyte-sourced myeloperoxidase, may lead to atrial fibrogenesis and extracellular matrix metamorphosis through matrix metalloproteinases (57, 58). With the rapid aging of the population, the confluence of atrial fibrillation and cancer-related complications is becoming increasingly significant and prevalent in clinical settings, particularly in low- and middle-income countries with weaker healthcare and economic infrastructures (59). Given the escalating incidence of thromboembolic events in atrial fibrillation patients, there is an associated poorer prognosis for newly diagnosed atrial fibrillation in the context of cancer (60). On the other hand, initiating anticoagulant therapy after the diagnosis of atrial fibrillation may serve as an indicator of occult cancer, especially in gastrointestinal sites, as manifested by bleeding warning signs (59). Henceforth, it may be warranted to implement more intensive surveillance for potential malignancies within the digestive system among patients with AF, aiming to optimize prognostication, assessment, and prevention of cardiovascular complications.

Contemporary epidemiological inquiries and case-control examinations indicate that individuals with extant HF and roughly 4% of those enduring IS exhibit a heightened likelihood for subsequent malignancy onset (61, 62). In a distinct community-oriented cohort, HF-afflicted subjects bore an amplified oncogenic risk, notwithstanding age or gender considerations (63). One analytical pursuit projected a 20% surge in age-normalized oncogenic onset a year post-IS (64). The physiological intricacies anchoring the ties between acute ischemic stroke and oncogenesis remain nebulous (65). Importantly, the aforementioned investigations predominantly proffer associative insights, lacking a causative underpinning. In contrast, our analysis evidences HF as a mediating agent between AF and malignancies of the esophagus and small intestine, and IS as an intermediary between AF and colon cancer. On the flip side, preliminary indications suggest that AF might intrinsically heighten oncogenic diagnosis susceptibility (5, 66). Yet, the contention of whether this correlation is a manifestation of detection skewness or a genuine causal liaison remains a matter of ongoing discourse (8). The results and mechanistic interpretations from the previously referenced observational investigations align consistently with our findings.

Our insights are predicated upon a confluence of advancements: initially, the availability of extensive phenotyping of GWAS confers a sufficiently voluminous array of genetic instruments to facilitate the identification of robust genetic variants conducive to MR scrutiny of each disease. These bedrock initiatives capacitate a more stringent comparative appraisal of traits within univariate and mediator MR frameworks. Additionally, utilizing summary statistics from extensive GWAS for exposures, mediators, and outcomes enhances the statistical analysis power, supporting our hypothesis. Techniques such as MR-Egger and weighted median MR can furnish credible evidence of causality (67), notwithstanding the presence of confounding due to unbalanced pleiotropic influences. Within the purview of our investigation, the univariate MR outcomes bolster the thesis that AF plays a cardinal role among the cancers mentioned above, and mediated MR results suggested a unique reciprocal risk factor relationship with HF and IS.

Parallel to the inherent constraints of our undertaking, several pivotal facets warrant underscoring. We employed an MR model to contrast outcomes across genotypes, analogous to the dichotomy seen in randomized controlled trials between intervention and control groups. Yet, caution is warranted in drawing inferences, as genetically driven alterations in risk differ from those induced by interventions such as dietary changes (68). Our study pioneers the use of two-step MR to elucidate the relationship between AF and susceptibility to digestive cancers. Additionally, HF and IS emerge as potential mechanisms bridging the gap between atrial fibrillation and digestive malignancies. While the European ancestry of our participant cohort contributes to ancestral homogeneity, the applicability of our findings to wider ethnographic spectrums is limited. Further investigations involving diverse ethnic populations are imperative to confirm these findings and uncover additional clinical implications. Although horizontal pleiotropy can be investigated or corrected using methods such as MR-Egger or the IVW method of Cochran’s Q test, these methods typically require a substantial number of instrumental SNPs. Due to the limited quantity and potency of the genetic instruments’ SNPs, caution should be exercised in the interpretation of certain conclusions drawn in this study. In future research, acknowledging the critical role of sample size constraints is pivotal. It is imperative to explore collaborations and acquire larger datasets to fortify the robustness and generalizability of upcoming studies. In culmination, our analytical profundity rests upon substantial cohorts extracted from repositories like UKBB and FinnGen, buttressed by supplementary research endeavors (20, 29).

## Conclusion

In summary, our findings indicate that AF is a pivotal causal factor in the predisposition to esophageal, colon, and small intestine neoplasms, with IS serving as a mediator in the etiology of colon cancer and HF coordinating similar processes for both esophageal and small intestine malignancies; other associations did not reach statistical significance. This research enhances understanding of the complex relationship in cardiovascular oncology, offering a potential prophylactic direction to address the rising global burden of AF and digestive malignancies, championing a shift toward salubrious living.

## Data availability statement

The original contributions presented in the study are included in the article/**Supplementary Material**. Further inquiries can be directed to the corresponding authors.

## Ethics statement

Access to genetic and phenotypic data from UK Biobank and FinnGen is granted through a formal application and approval process.

## Author contributions

ZX: Conceptualization, Data curation, Formal analysis, Funding acquisition, Investigation, Methodology, Software, Validation, Visualization, Writing – original draft, Writing – review & editing. XR: Conceptualization, Data curation, Formal analysis, Funding acquisition, Investigation, Methodology, Visualization, Writing – original draft, Writing – review & editing. YX: Data curation, Writing – review & editing. ZZ: Data curation, Formal analysis, Writing – review & editing. LY: Formal analysis, Writing – review & editing. JH: Writing – review & editing. JZ: Conceptualization, Supervision, Writing – review & editing. RZ: Conceptualization, Supervision, Writing – review & editing.

## Supplementary material

Supplementary File 1 Scatter diagrams.

Supplementary File 2 ‘leave-one-out’ explorations.

Supplementary File 3 isolated SNP assessments.
