# Supplementary material for: Connecting atrial fibrillation to digestive neoplasms: exploring mediation via ischemic stroke and heart failure in Mendelian randomization studies
Source: Front Oncol. 2024 Feb 20;14:1301327. doi: 10.3389/fonc.2024.1301327 (PMC10912520; doi:10.3389/fonc.2024.1301327)
Supplement: Supplementary File 1 — Scatter diagrams. [file DataSheet_1.zip › File S2.PDF]

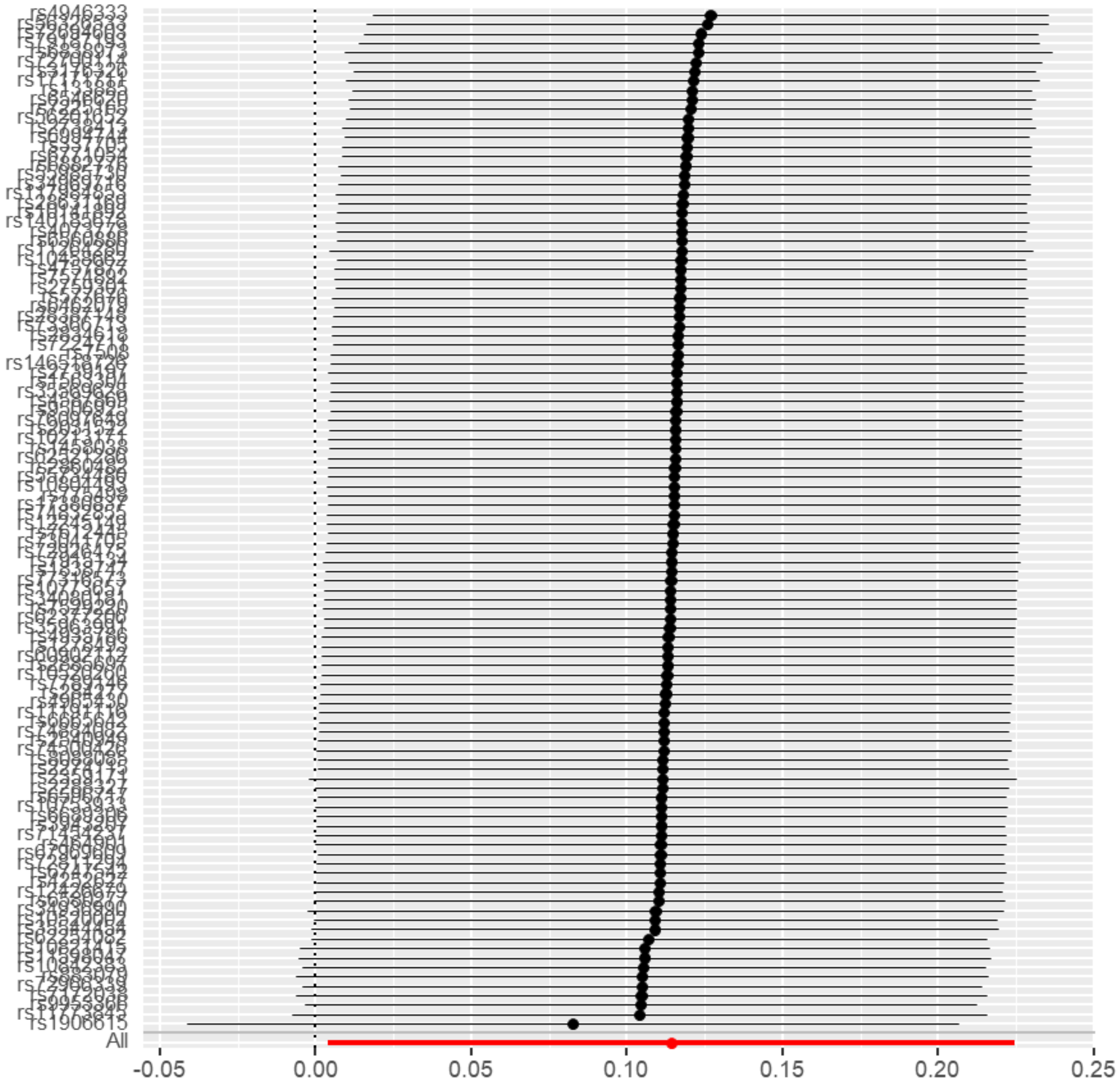

MR leave-one-out sensitivity analysis for
'Atrial fibrillation || id:ebi-a-GCST006414' on 'Malignant neoplasm of colon (all cancers excluded) || id:finn-b-C3_COLON_EXALLC'

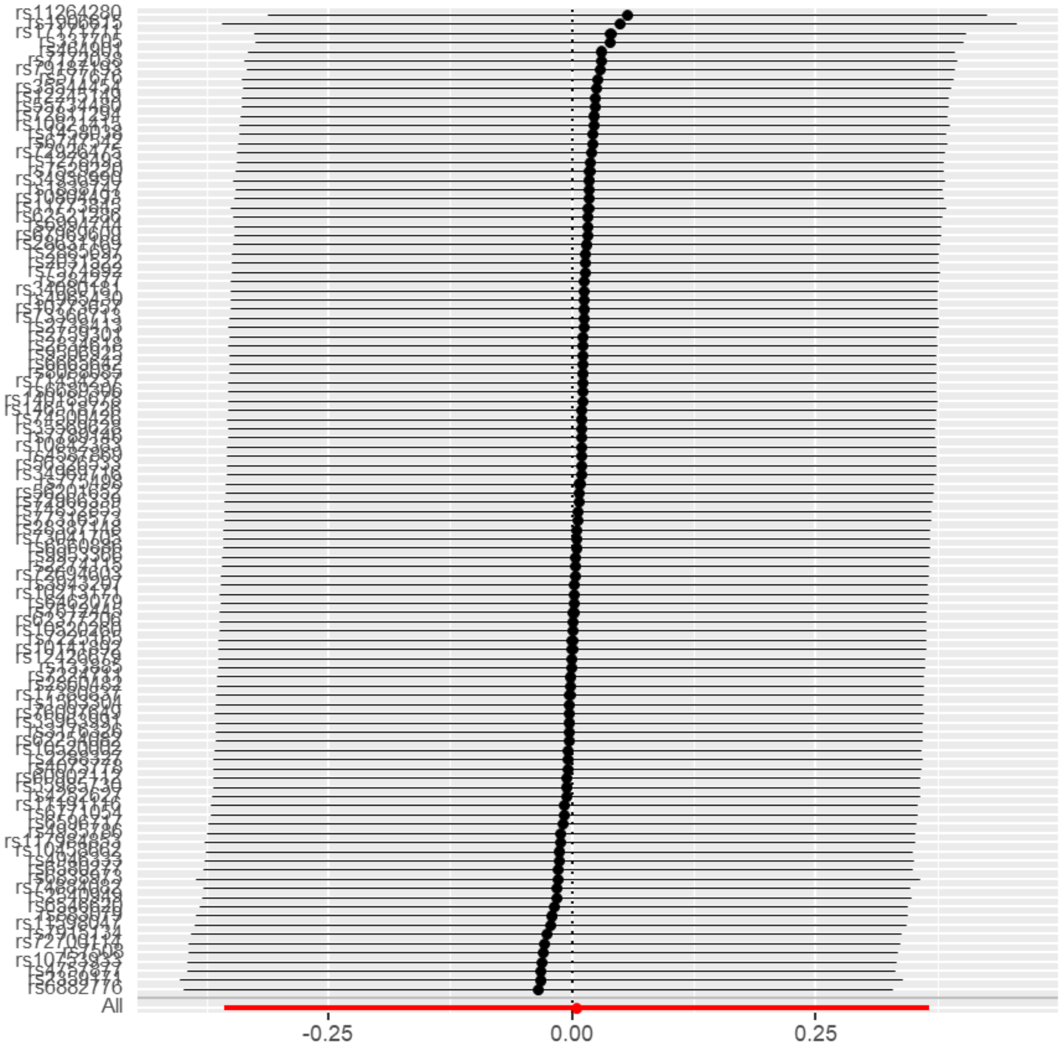

MR leave-one-out sensitivity analysis for
'Atrial fibrillation || id:ebi-a-GCST006414' on '"Malignant neoplasm of lip, oral cavity and pharynx" (all cancers excluded) || id:finn-b-C3_LIP_ORAL_PHARYNX_EXALLC'

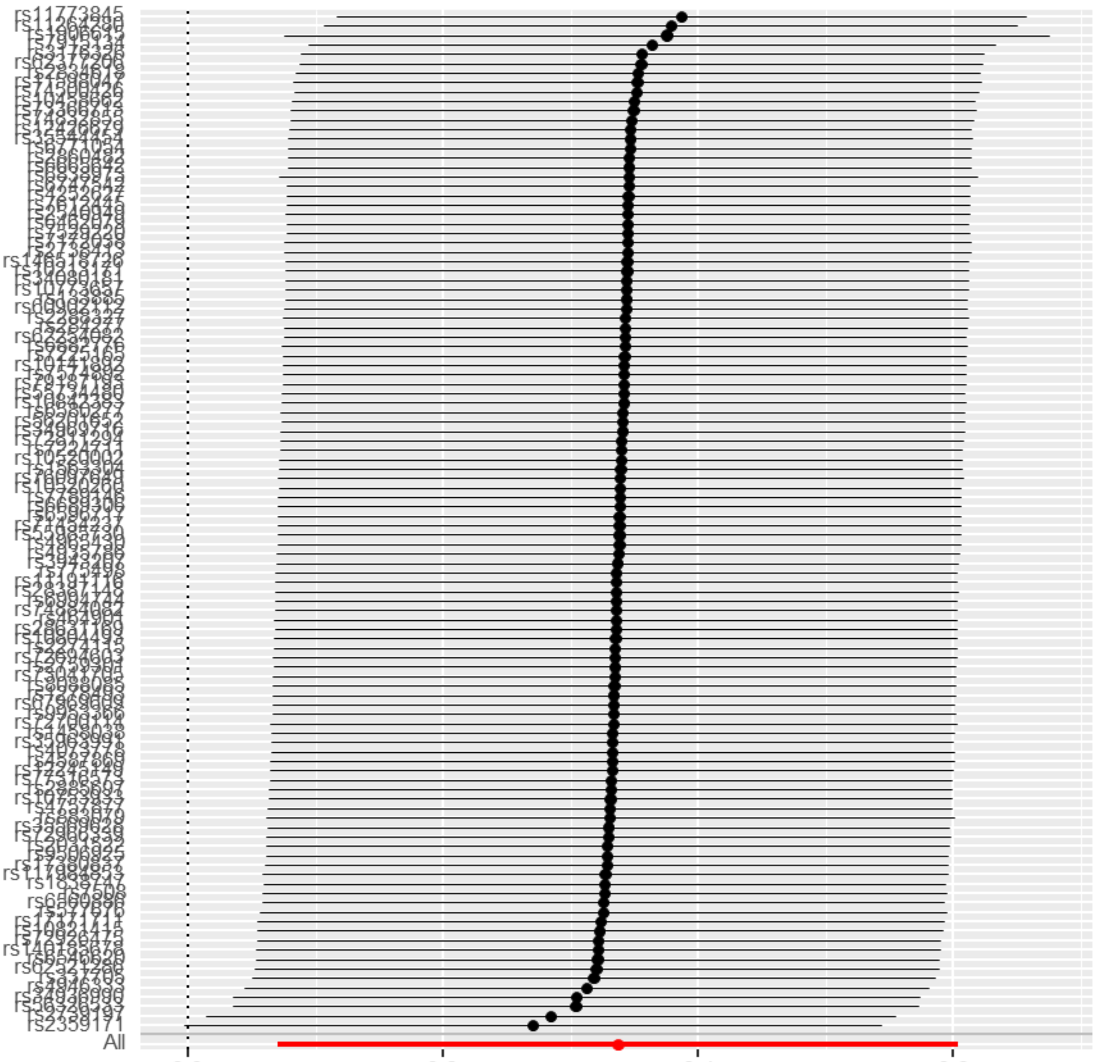

MR leave-one-out sensitivity analysis for
'Atrial fibrillation || id:ebi-a-GCST006414' on 'Malignant neoplasm of oesophagus (all cancers excluded) || id:finn-b-C3_OESOPHAGUS_EXALLC'

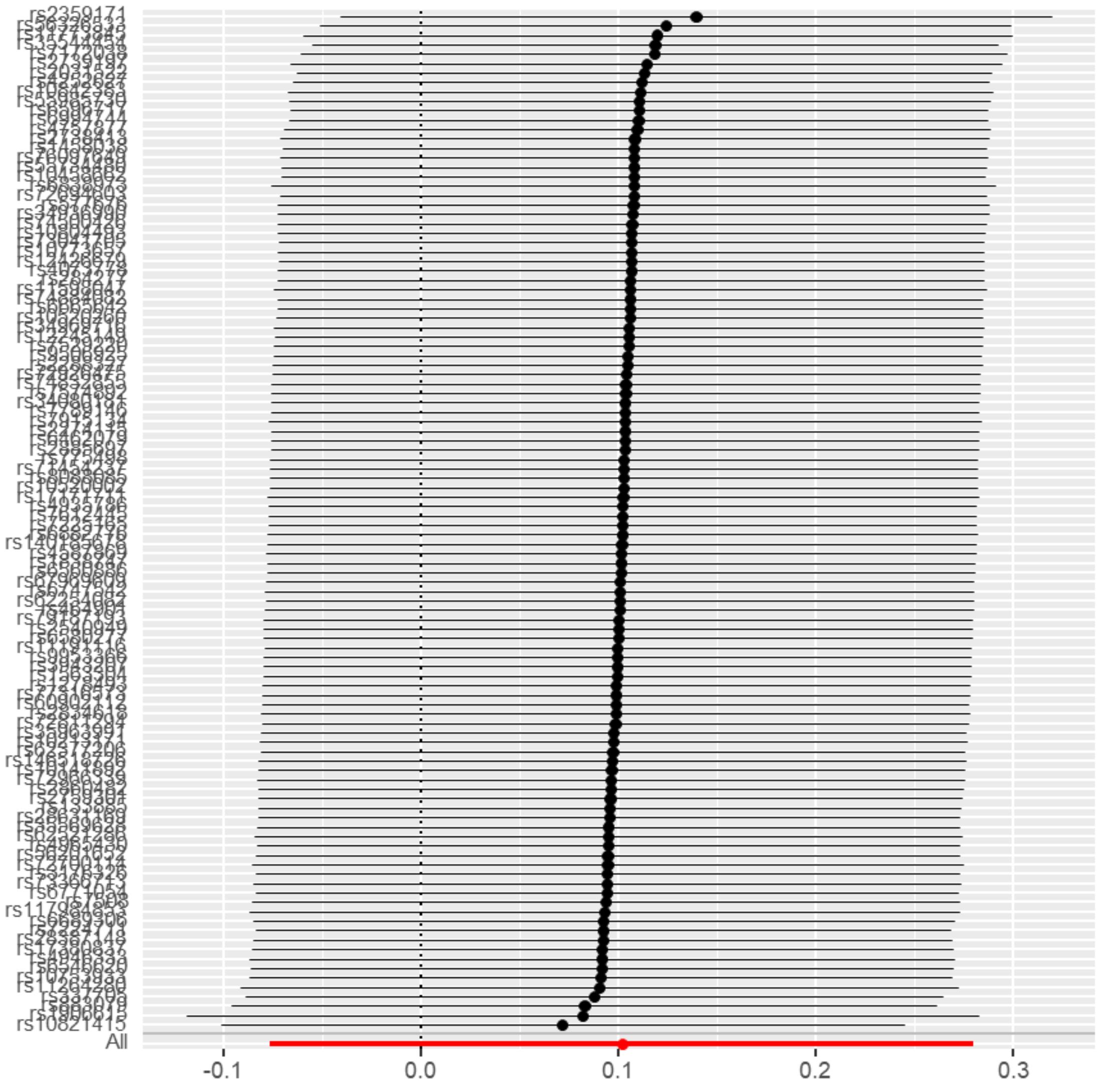

MR leave-one-out sensitivity analysis for
'Atrial fibrillation || id:ebi-a-GCST006414' on 'Malignant neoplasm of stomach (all cancers excluded) || id:finn-b-C3_STOMACH_EXALLC'

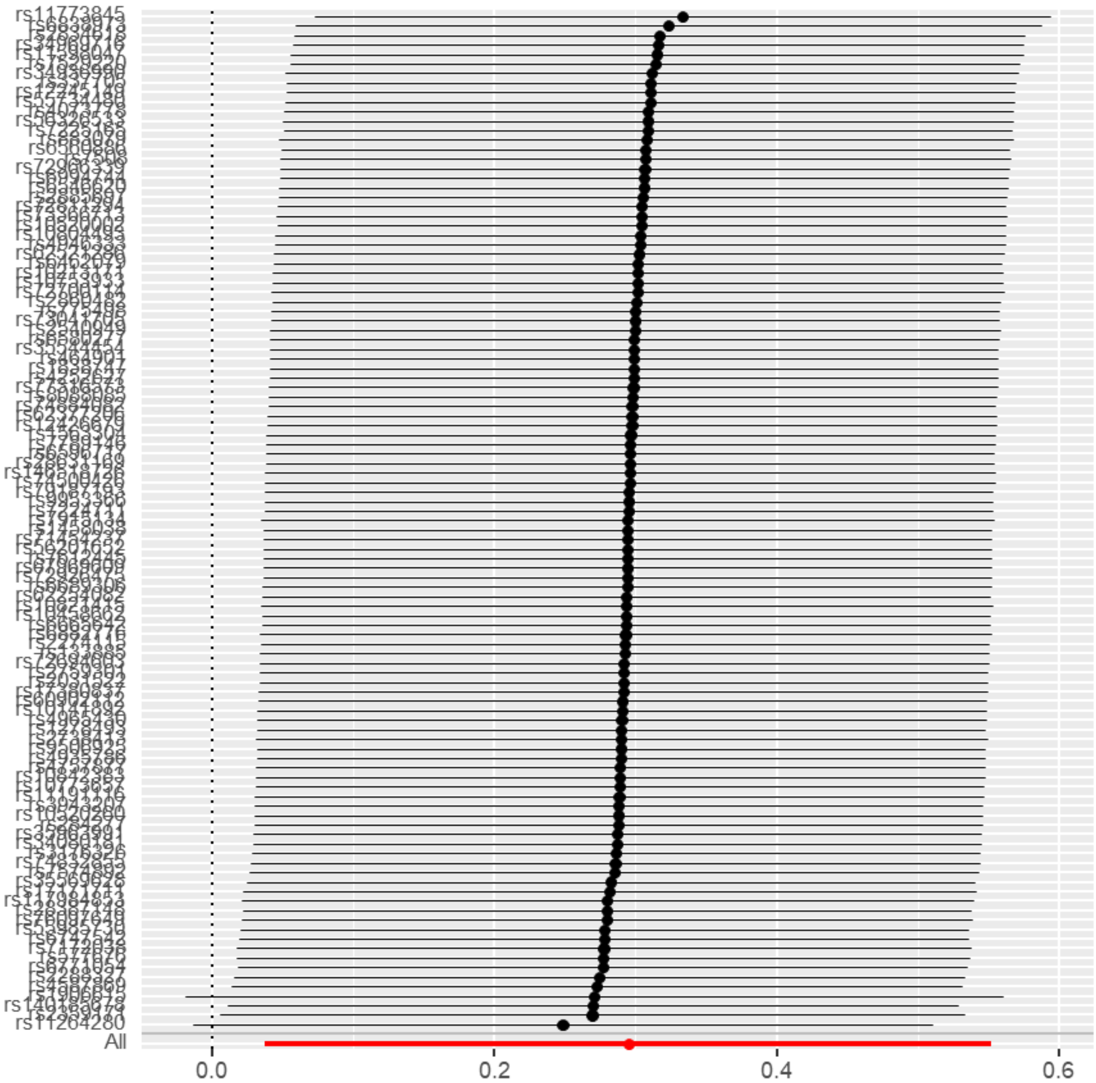

MR leave-one-out sensitivity analysis for
'Atrial fibrillation || id:ebi-a-GCST006414' on 'Malignant neoplasm of small intestine (all cancers excluded) || id:finn-b-C3_SMALL_INTESTINE_EXALLC'

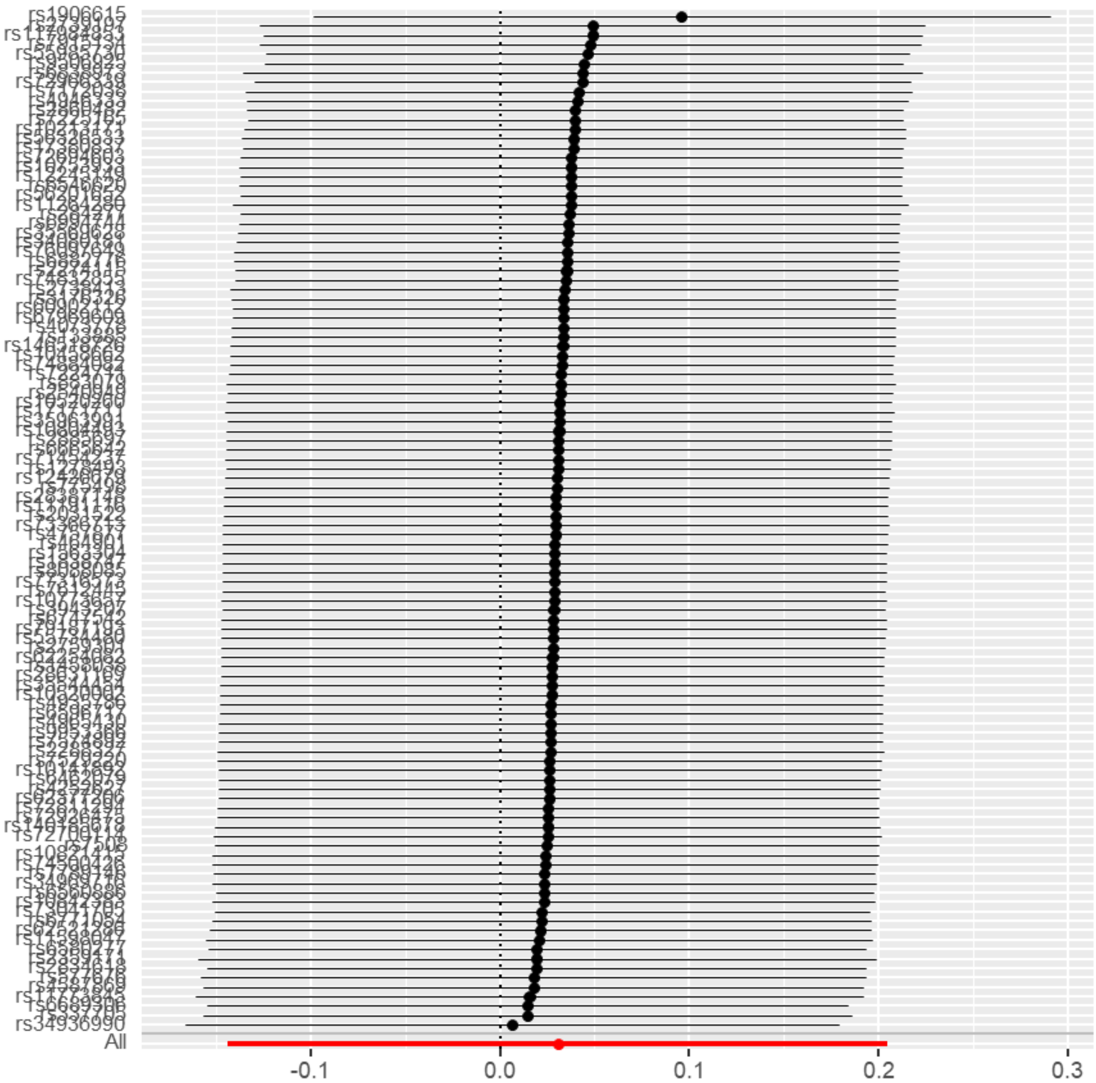

MR leave-one-out sensitivity analysis for
'Atrial fibrillation || id:ebi-a-GCST006414' on 'Malignant neoplasm of pancreas (all cancers excluded) || id:finn-b-C3_PANCREAS_EXALLC'

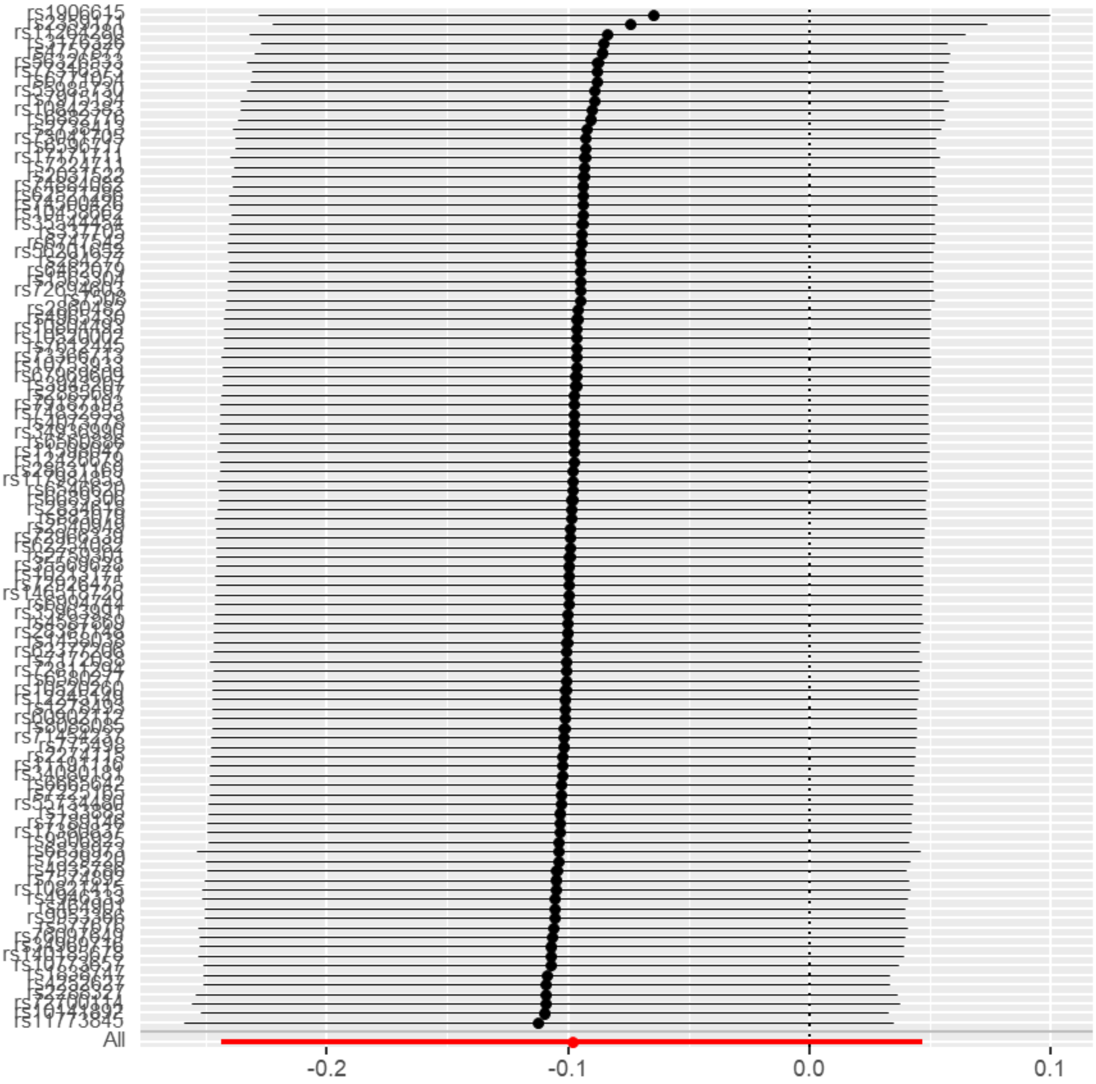

MR leave-one-out sensitivity analysis for
'Atrial fibrillation || id:ebi-a-GCST006414' on 'Malignant neoplasm of rectum (all cancers excluded) || id:finn-b-C3_RECTUM_EXALLC'
