# Supplementary figures and images for: Connecting atrial fibrillation to digestive neoplasms: exploring mediation via ischemic stroke and heart failure in Mendelian randomization studies
Source: Front Oncol. 2024 Feb 20;14:1301327. doi: 10.3389/fonc.2024.1301327 (PMC10912520; doi:10.3389/fonc.2024.1301327)

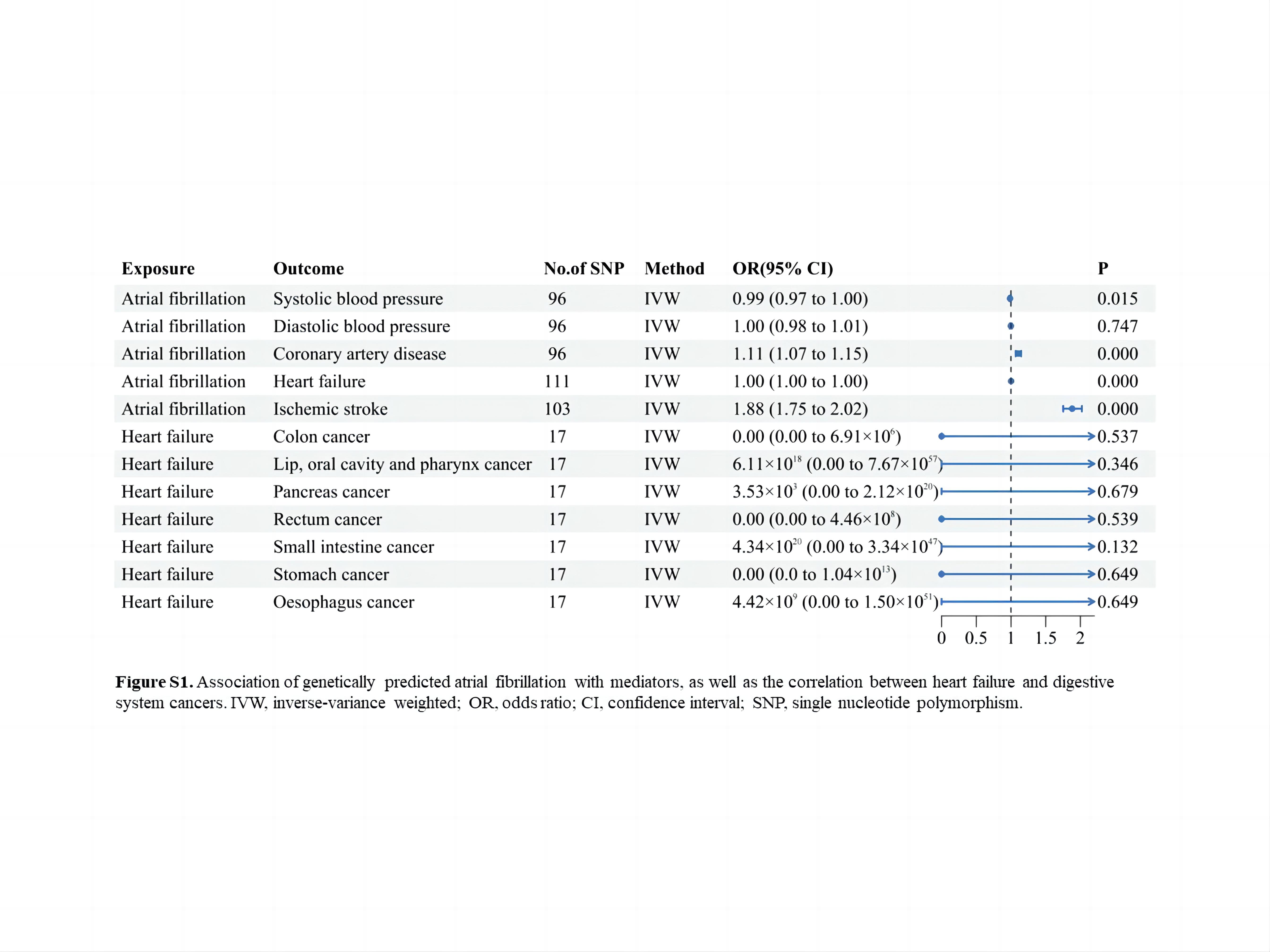

Supplement: Supplementary File 1 — Scatter diagrams. [file DataSheet_1.zip › Figure S1.JPEG]

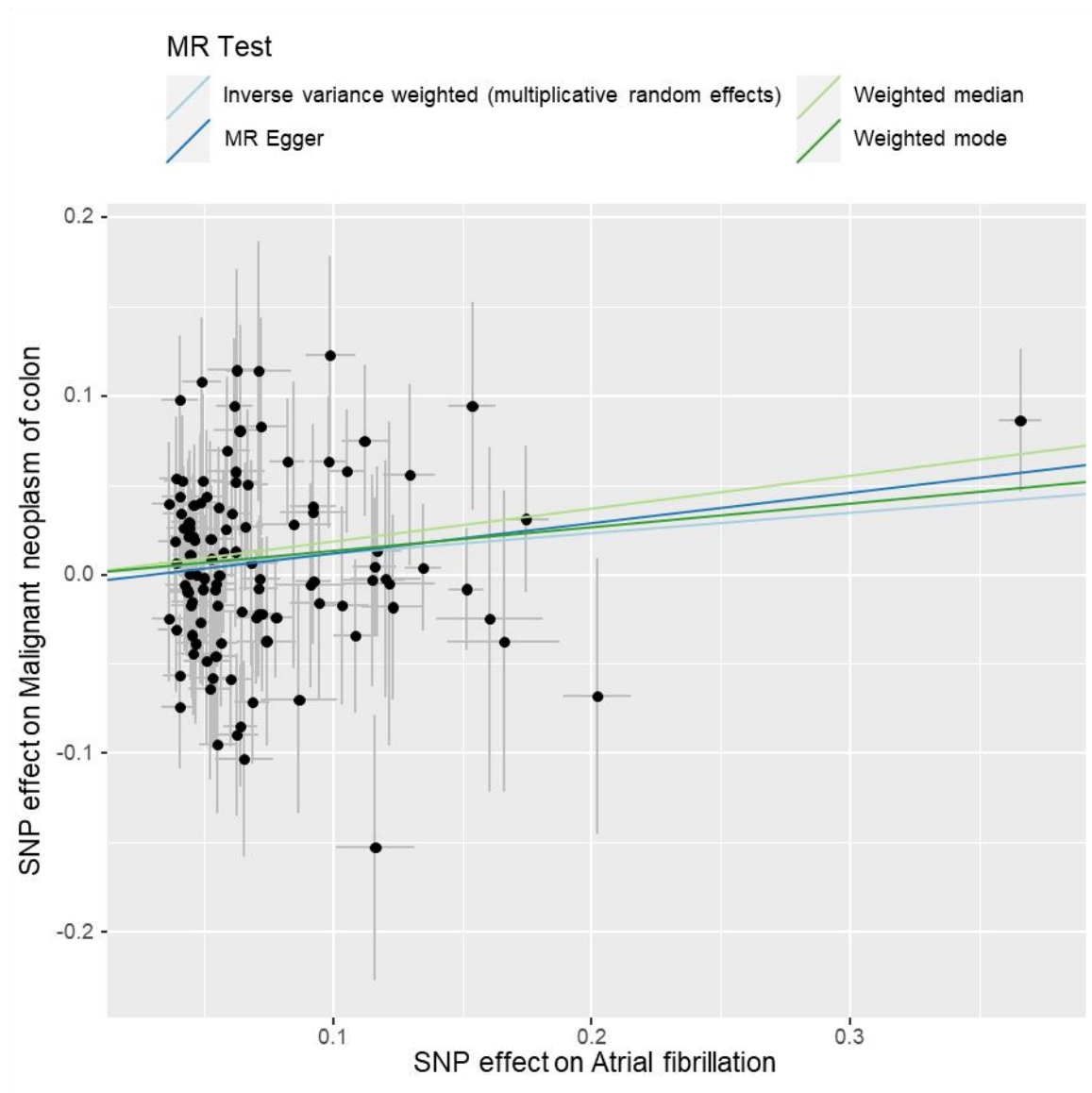

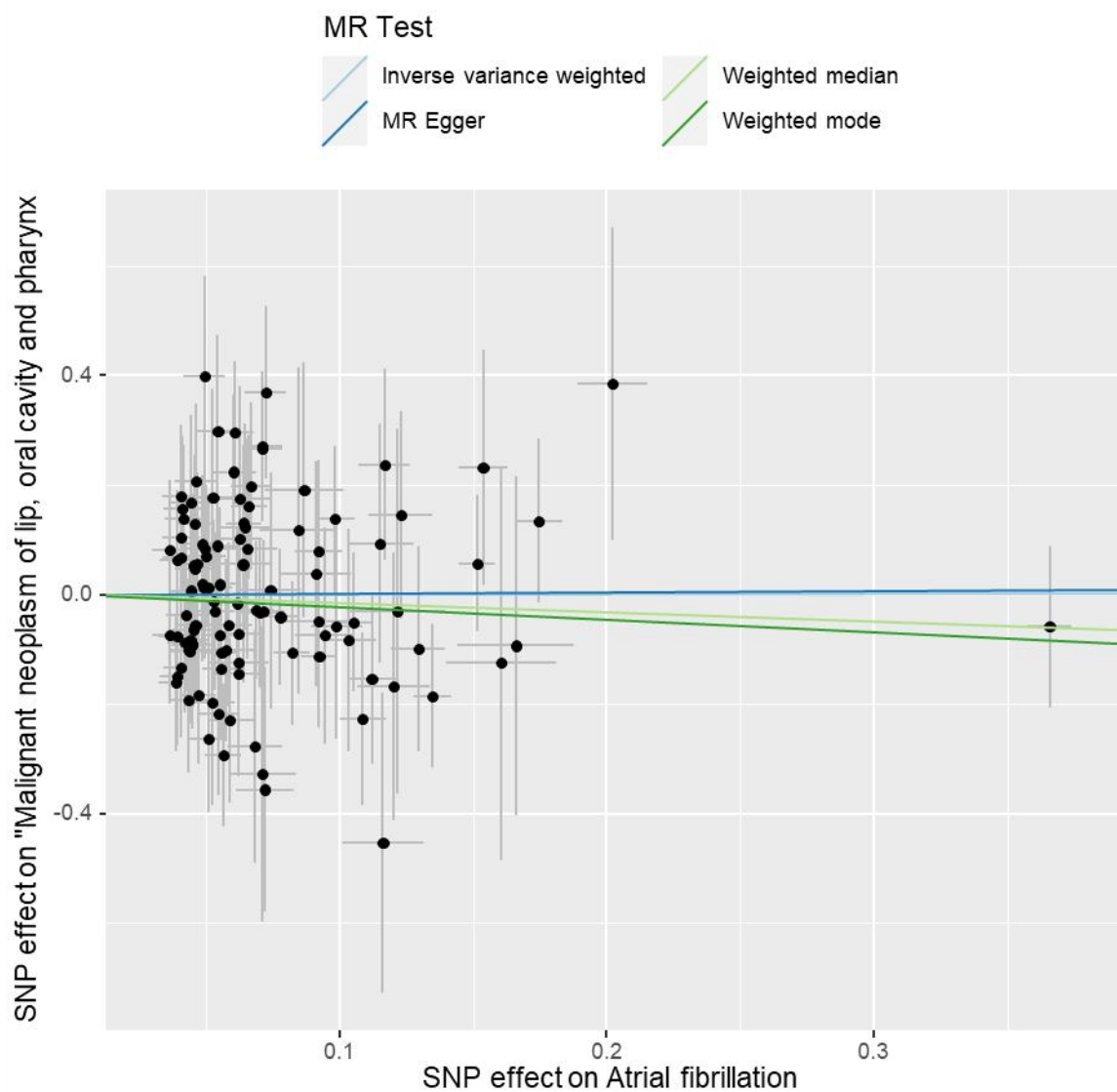

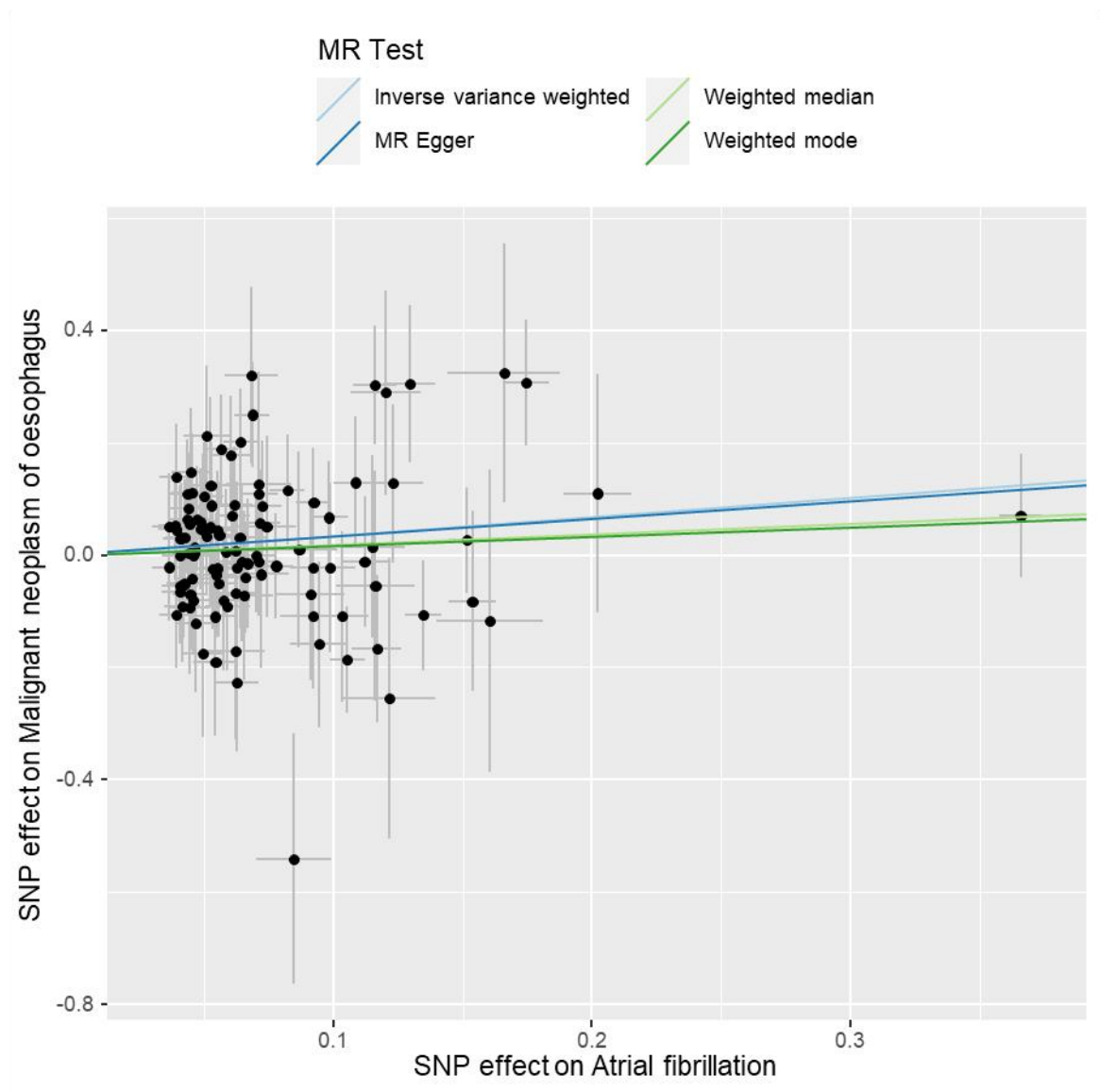

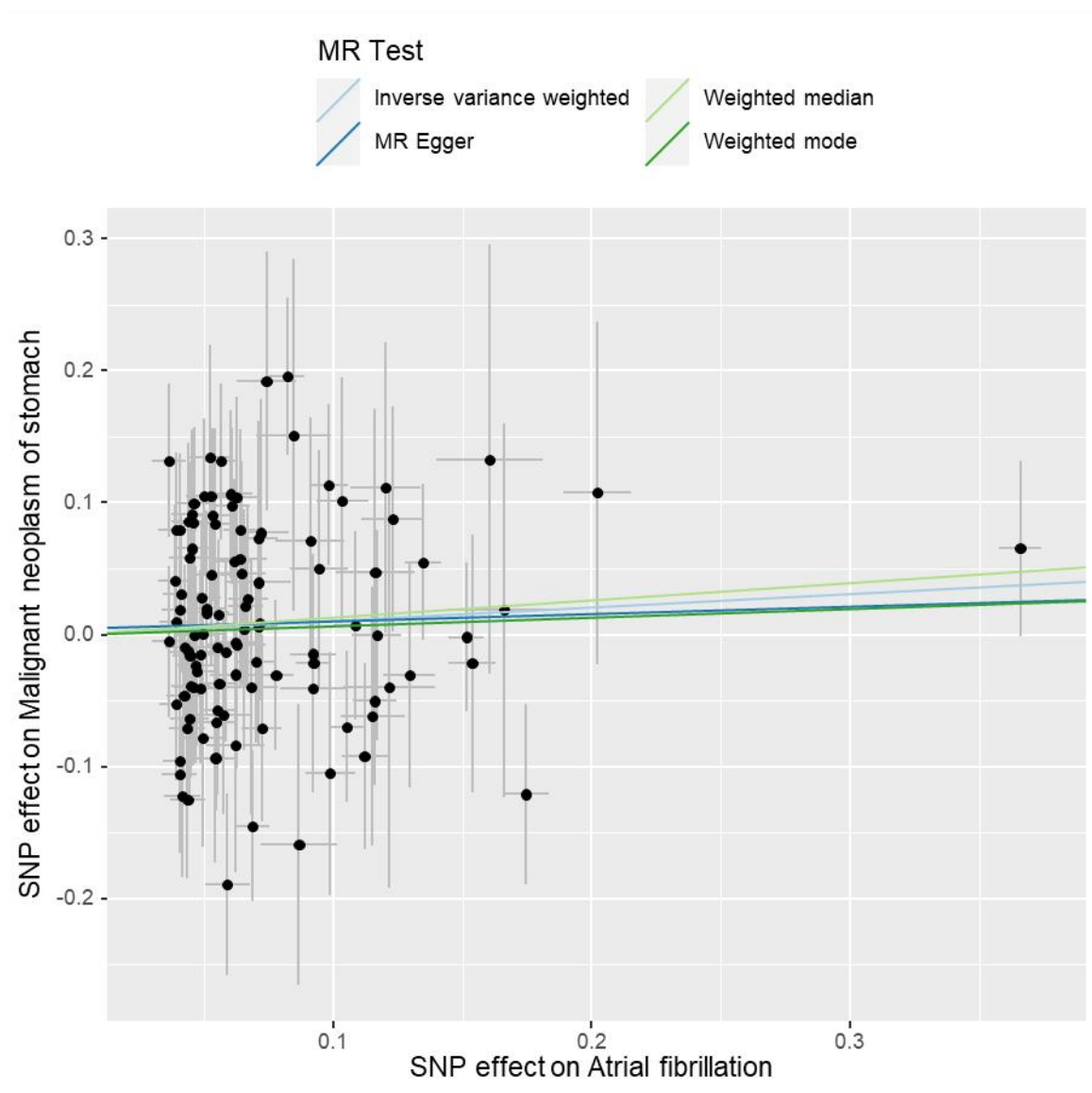

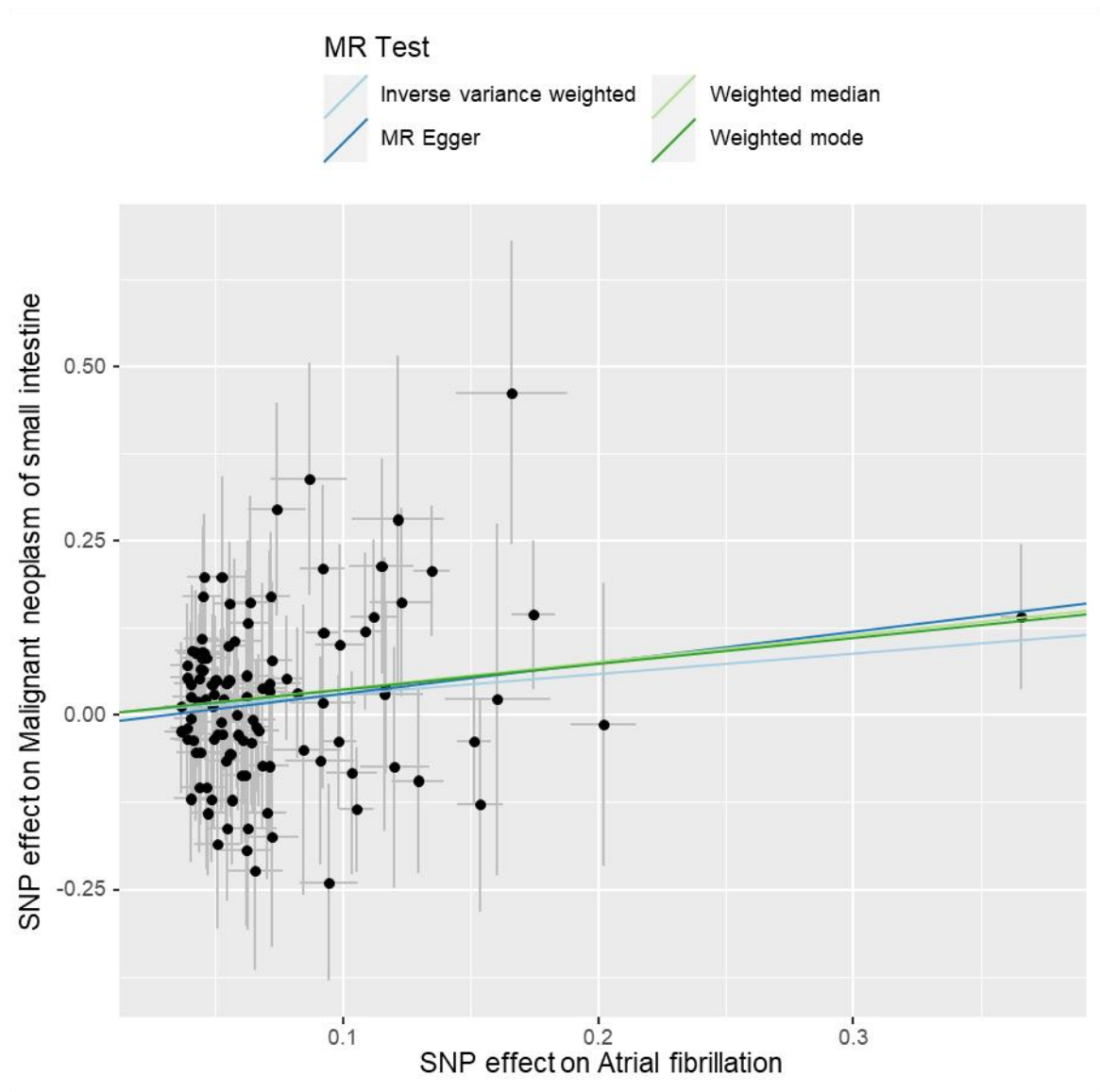

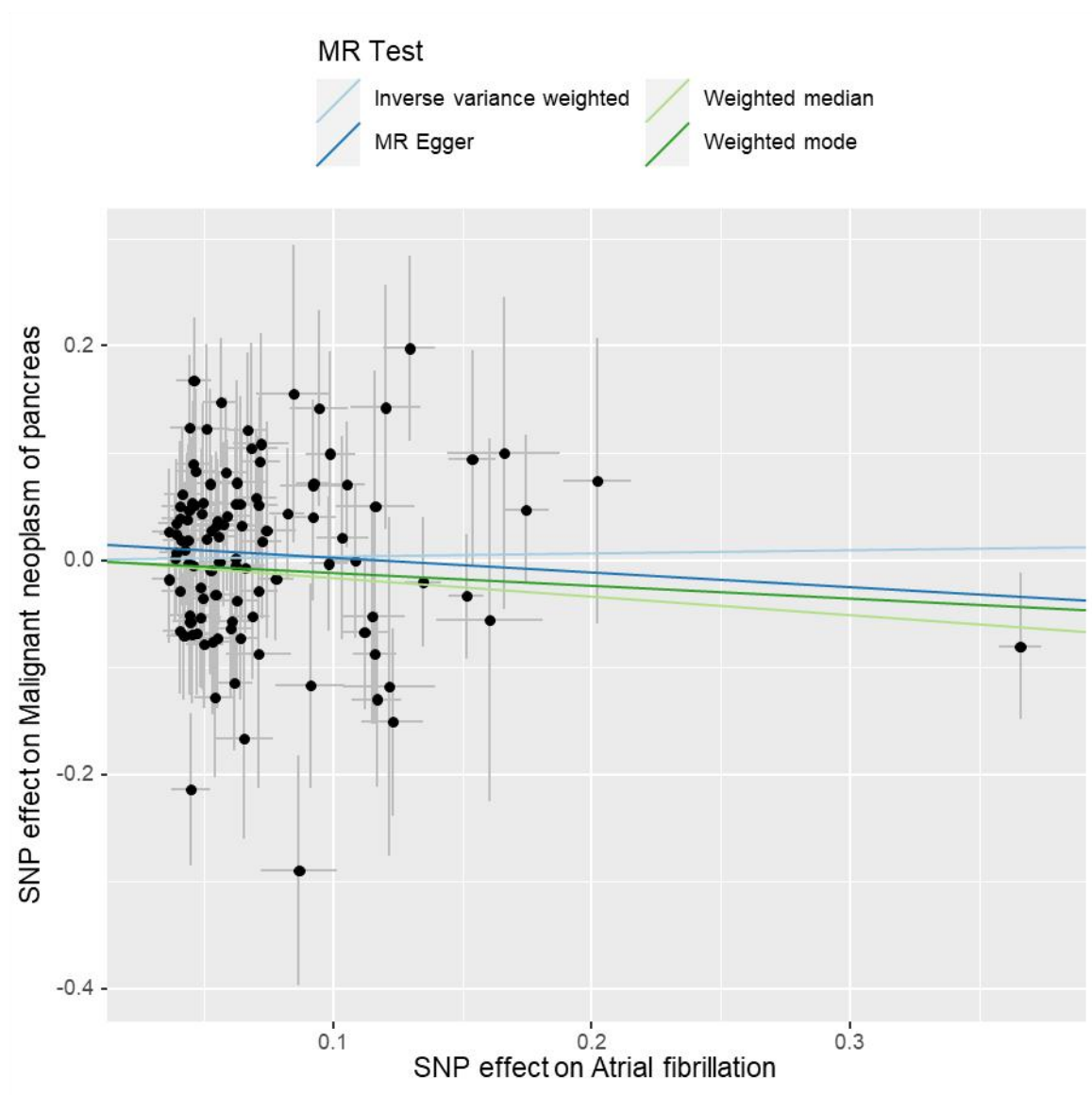

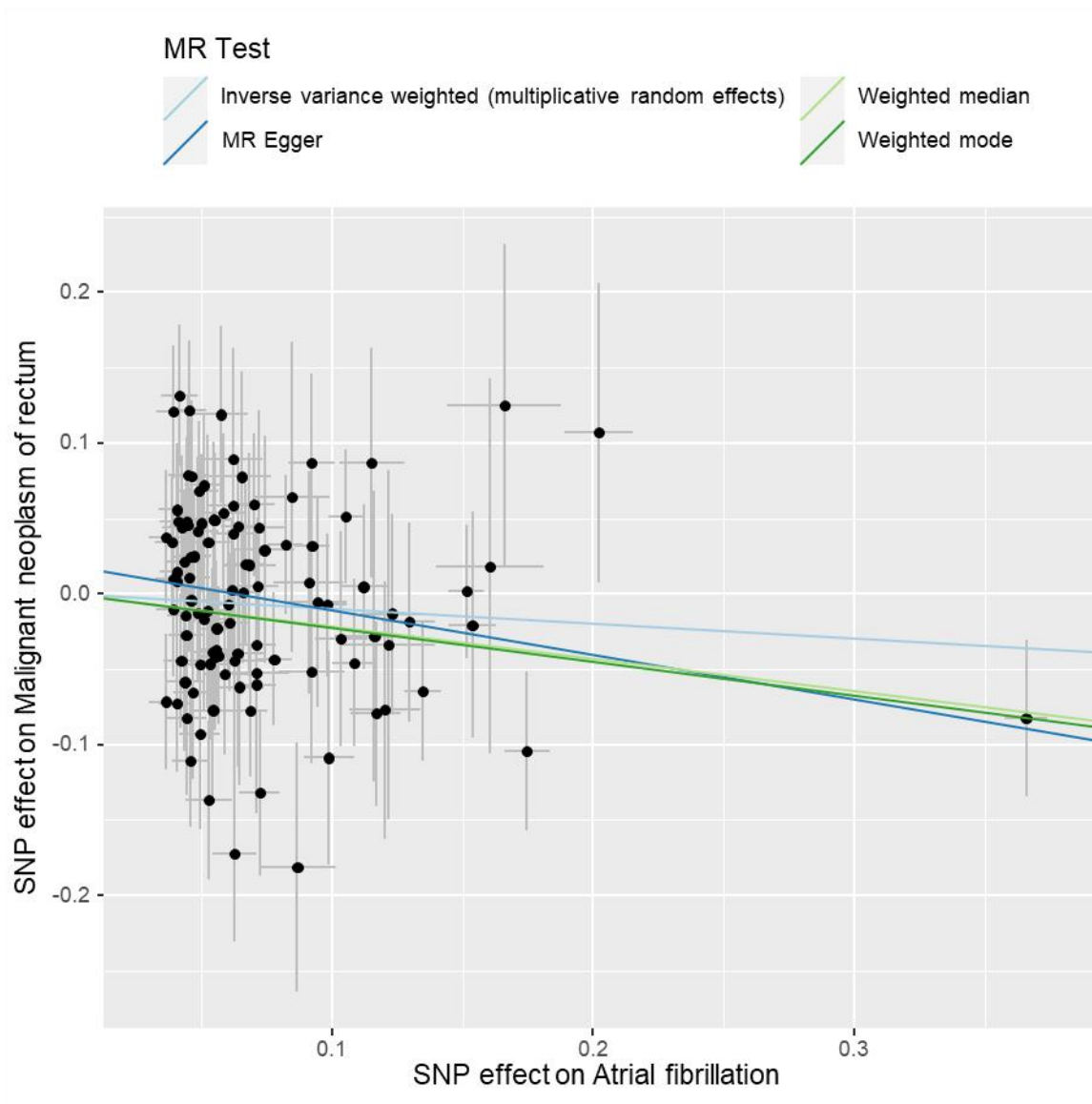

Supplement: Supplementary File 1 — Scatter diagrams. [file DataSheet_1.zip › File S1.PDF]

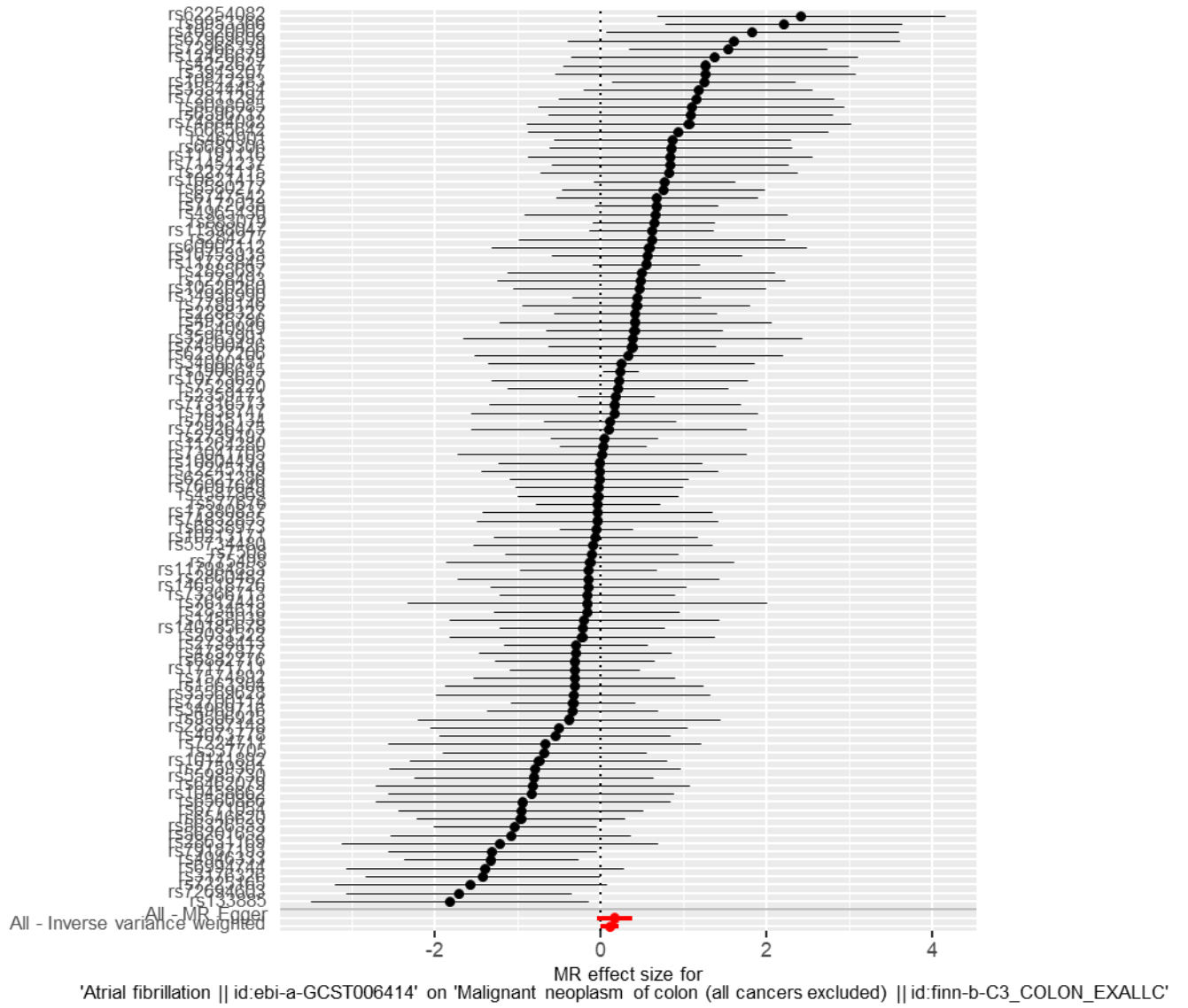

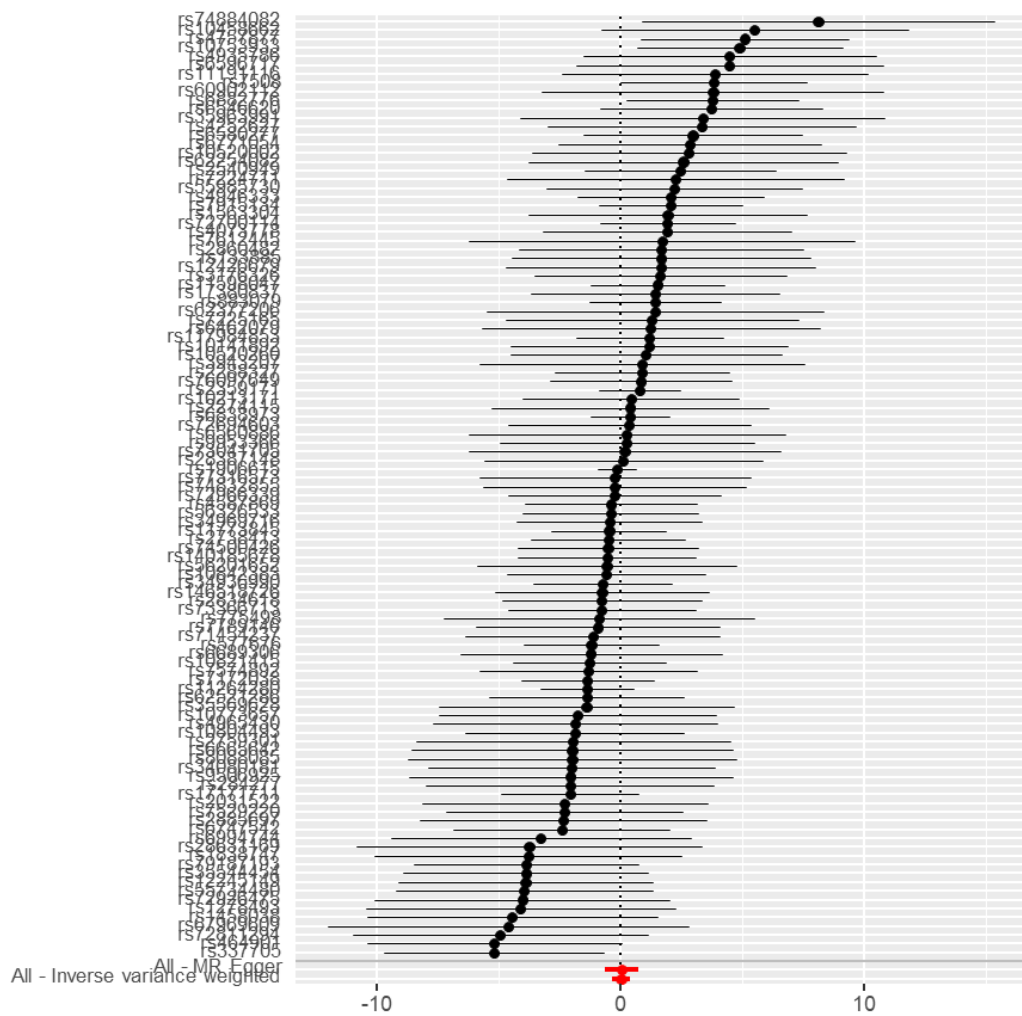

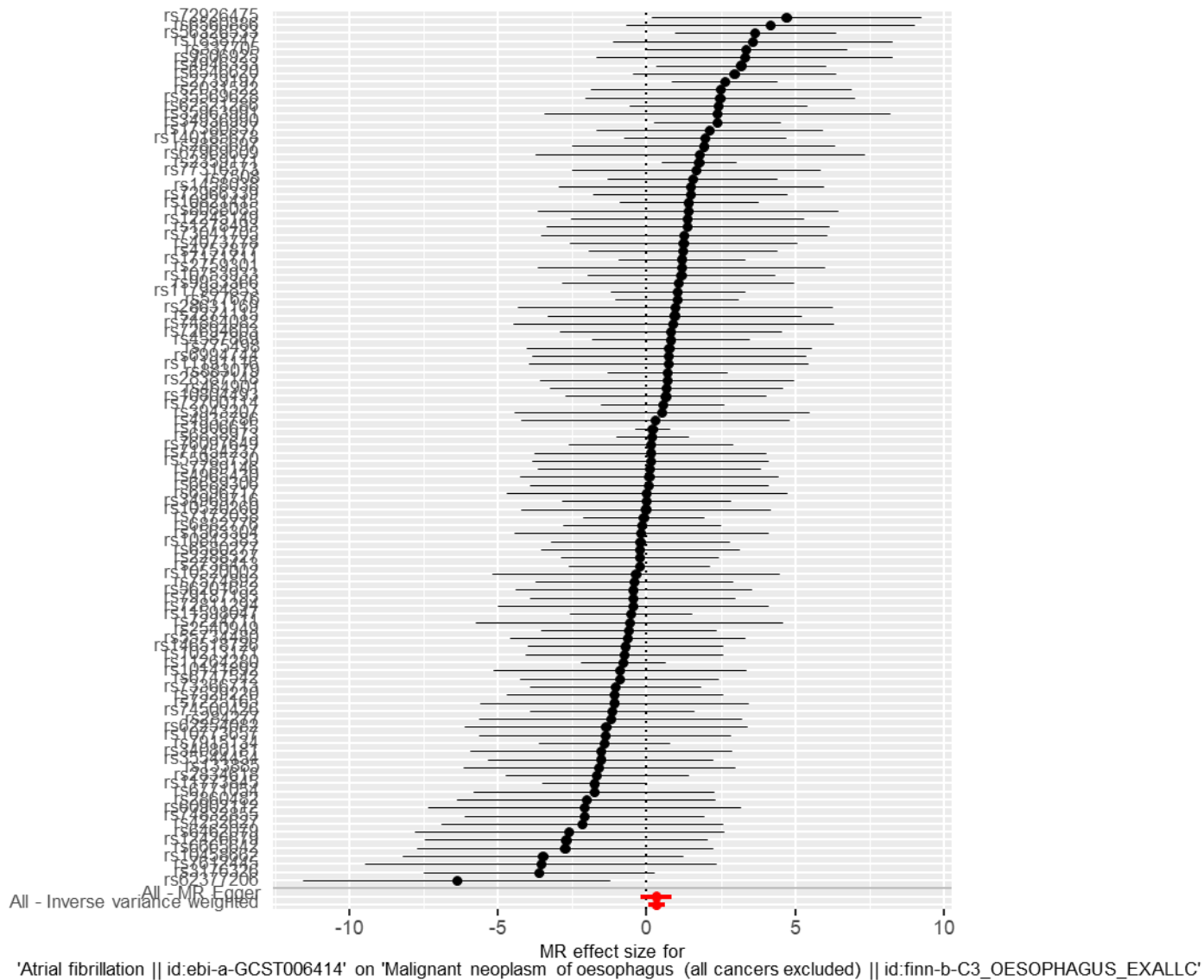

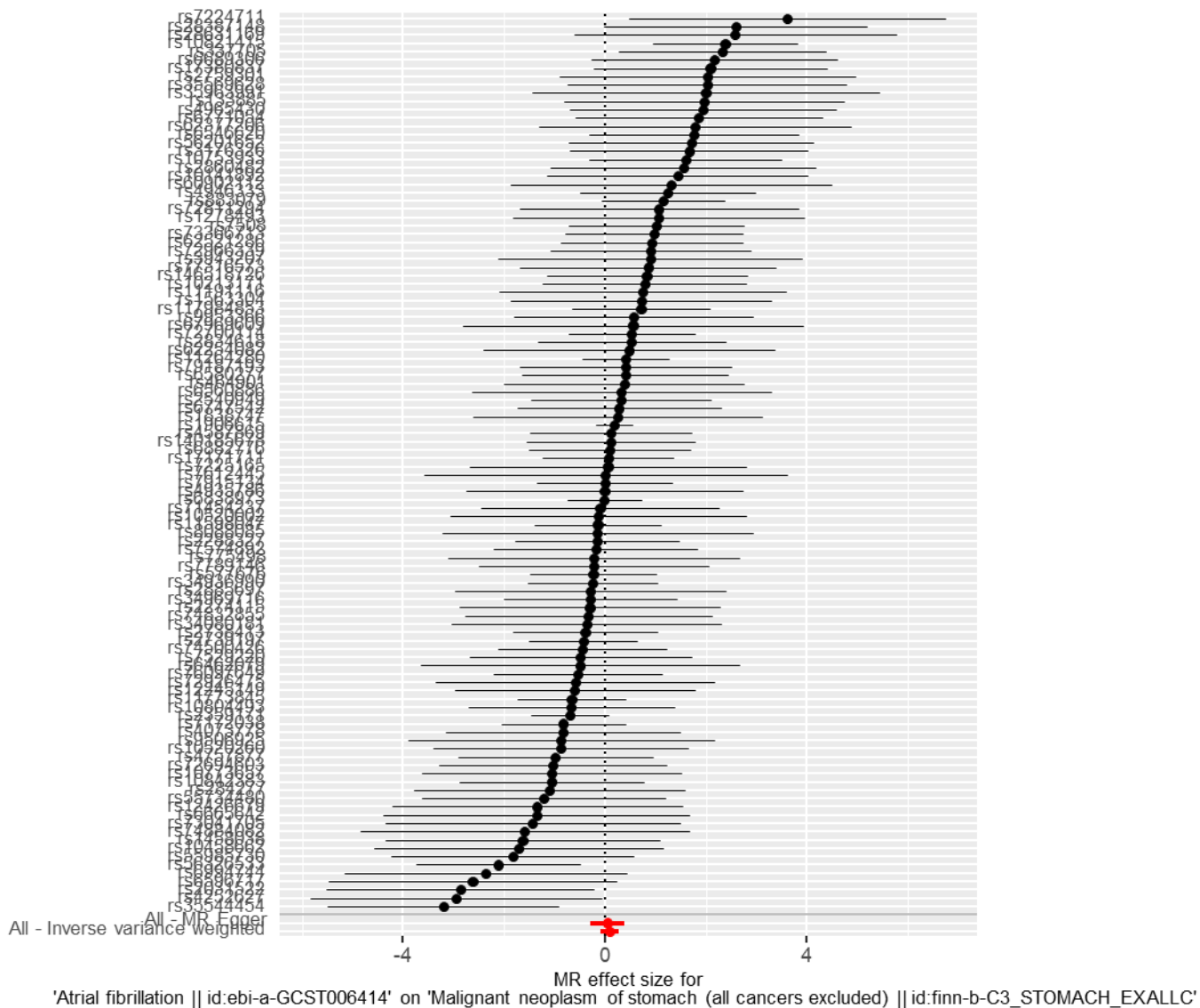

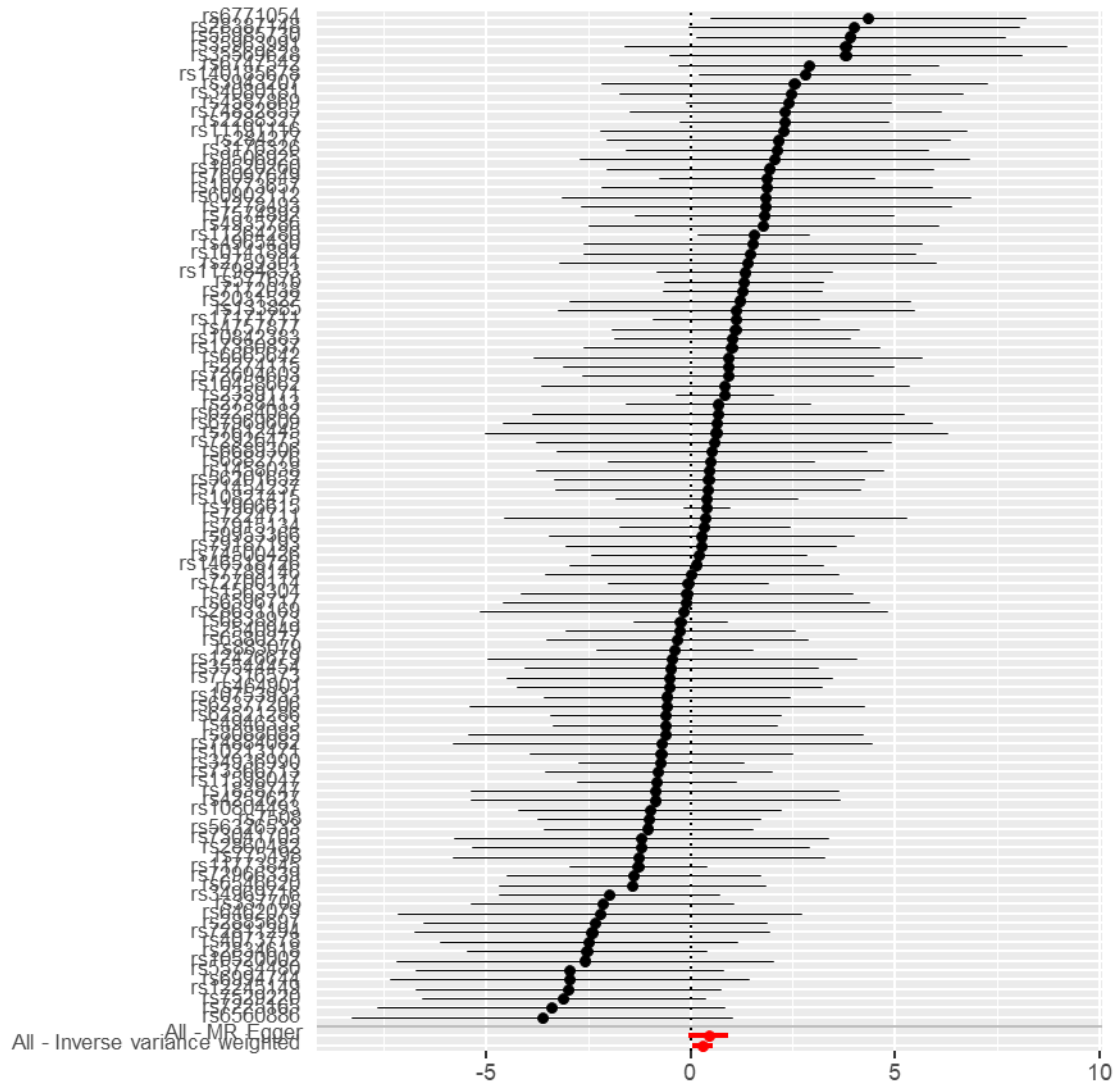

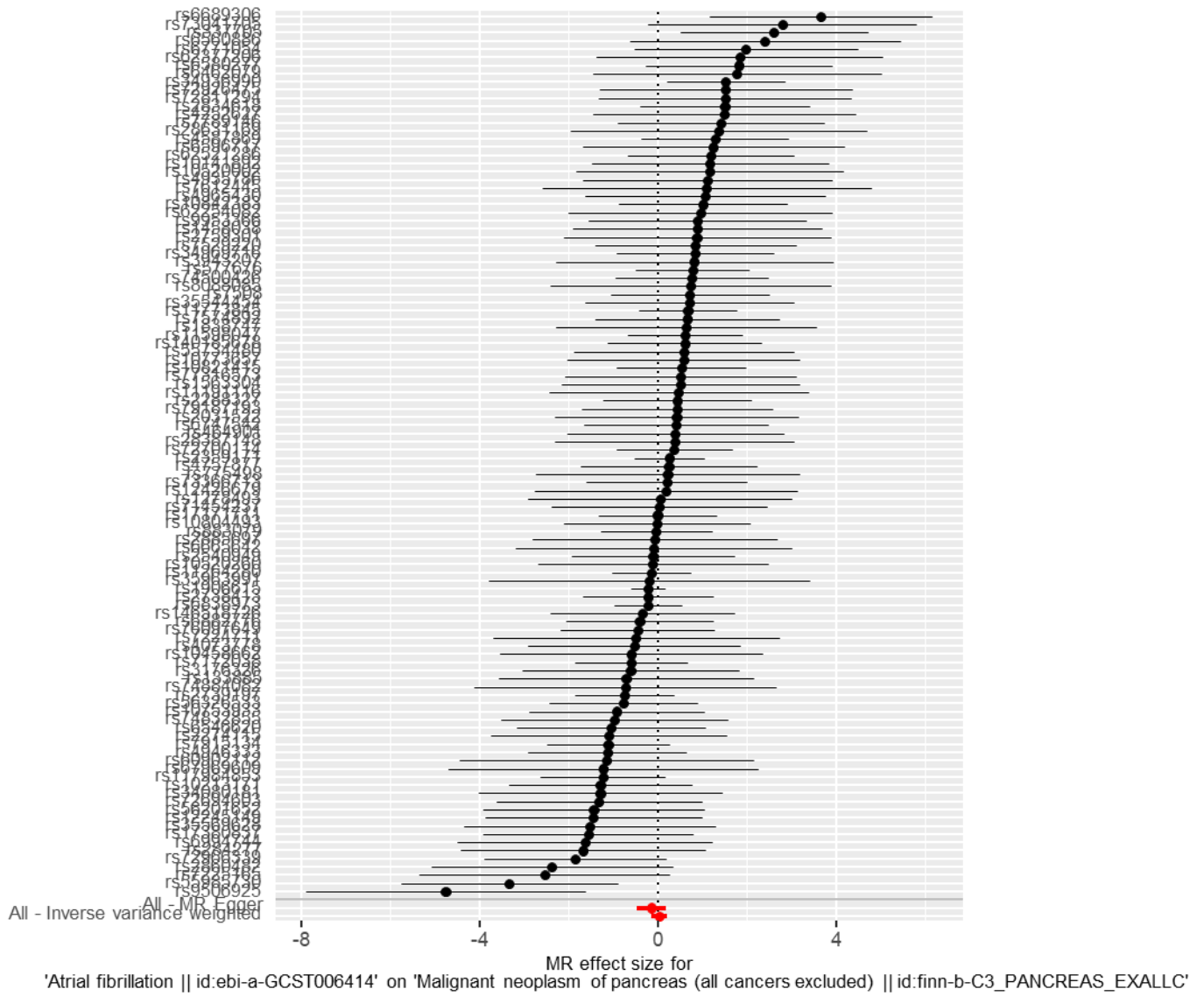

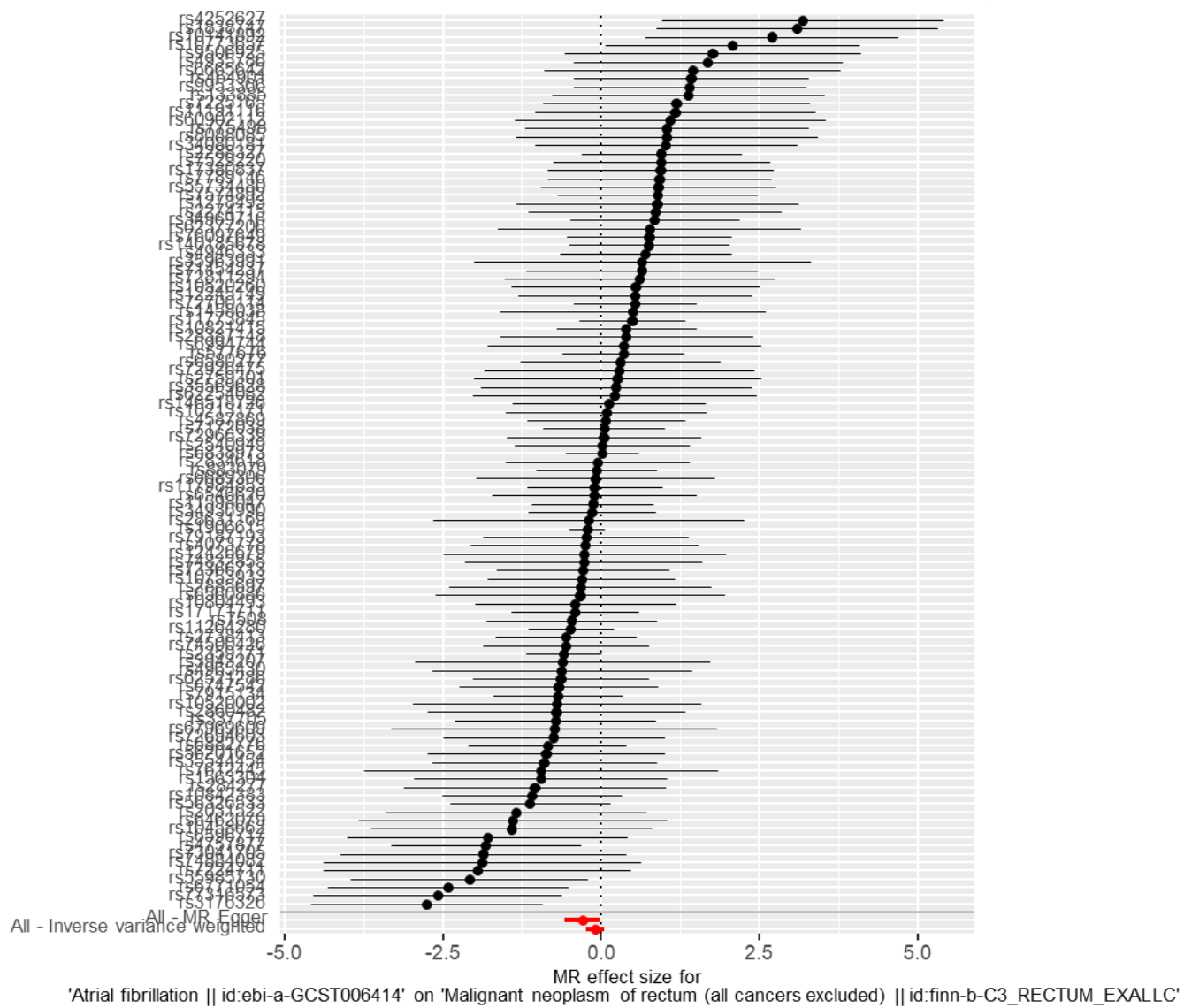

Supplement: Supplementary File 1 — Scatter diagrams. [file DataSheet_1.zip › File S3.PDF]
